# Scaffold-Hopping from Synthetic Drugs by Holistic Molecular Representation

**DOI:** 10.1038/s41598-018-34677-0

**Published:** 2018-11-07

**Authors:** Francesca Grisoni, Daniel Merk, Ryan Byrne, Gisbert Schneider

**Affiliations:** 1 Swiss Federal Institute of Technology (ETH), Department of Chemistry and Applied Biosciences, Vladimir-Prelog-Weg 4, CH-8093 Zurich, Switzerland; 2 Milano Chemometrics & QSAR Research Group, Department of Earth and Environmental Sciences, University of Milano-Bicocca, IT-20126 Milano, Italy

## Abstract

The discovery of novel ligand chemotypes allows to explore uncharted regions in chemical space, thereby potentially improving synthetic accessibility, potency, and the drug-likeness of molecules. Here, we demonstrate the scaffold-hopping ability of the new Weighted Holistic Atom Localization and Entity Shape (WHALES) molecular descriptors compared to seven state-of-the-art molecular representations on 30,000 compounds and 182 biological targets. In a prospective application, we apply WHALES to the discovery of novel retinoid X receptor (RXR) modulators. WHALES descriptors identified four agonists with innovative molecular scaffolds, populating uncharted regions of the chemical space. One of the agonists, possessing a rare non-acidic chemotype, revealed high selectivity on 12 nuclear receptors and comparable efficacy as bexarotene on induction of ATP-binding cassette transporter A1, angiopoietin like protein 4 and apolipoprotein E. The outcome of this research supports WHALES as an innovative tool to explore novel regions of the chemical space and to detect novel bioactive chemotypes by straightforward similarity searching.

## Introduction

Identifying novel isofunctional chemotypes of bioactive compounds is a key challenge in medicinal chemistry, to successfully explore uncharted regions in chemical space and improve synthetic accessibility, potency, or drug-likeness of hits and leads^1,2^. Ligand-based drug discovery has benefitted from the introduction of numerical representations of molecules (“molecular descriptors”)^3^ into computational workflows^4–6^. Molecular descriptors grasp different aspects of the molecular structure (*e.g*., presence of fragments^7,8^, distribution of pharmacophoric features^9^, atomic steric and electronic environment^10^), and have thus provided a sound basis for ligand-based virtual screening, target prediction efforts, and *de novo* design of small molecules^9,11–16^. Many of the utilized molecular representations in virtual screening emphasize descriptor comprehensibility (*e.g*., presence of fragments, molecular connectivity) and ease of calculation, potentially affecting their scaffold-hopping ability^17^ and applicability to the identification of novel chemotypes. Additionally, the continuously increasing number of molecular descriptors proposed in the scientific literature (*e.g*.^3,18–22^) makes it necessary to identify the optimal set of molecular descriptors to employ for each user-tailored application.

Recently, we have developed a novel molecular representation, the WHALES (*Weighted Holistic Atom Localization and Entity Shape*) descriptors^23^, which were originally designed to transfer relevant structural and pharmacophore information encoded in known bioactive natural products (NP) to synthetically more accessible isofunctional compounds through similarity-driven approaches. In the proof-of-concept study^23^, WHALES identified seven natural-product-inspired synthetic compounds that modulate the cannabinoid receptor, with innovative scaffolds compared to actives annotated in ChEMBL^24^.

The aim of the present study is to extend the analysis of WHALES descriptors beyond NP-related applications. Thus, we performed a systematic retrospective virtual screening, to (i) determine the scaffold-hopping ability of WHALES with synthetic compounds as queries, and (ii) compare the performance of WHALES with seven state-of-the-art molecular descriptors. In this context, WHALES confirmed to possess a desirable scaffold-hopping ability, outperforming the state-of-the-art methods in 89% of the tested biological receptors. The scaffold-hopping ability of WHALES was confirmed by a prospective, experimental application of WHALES in finding synthetic modulators of the retinoid X receptor (RXR), through the identification of four novel agonists, including a new non-acidic RXR agonist chemotype.

## Results and Discussion

### Weighted Holistic Atom Localization and Entity Shape (WHALES)

**Figure 1:**
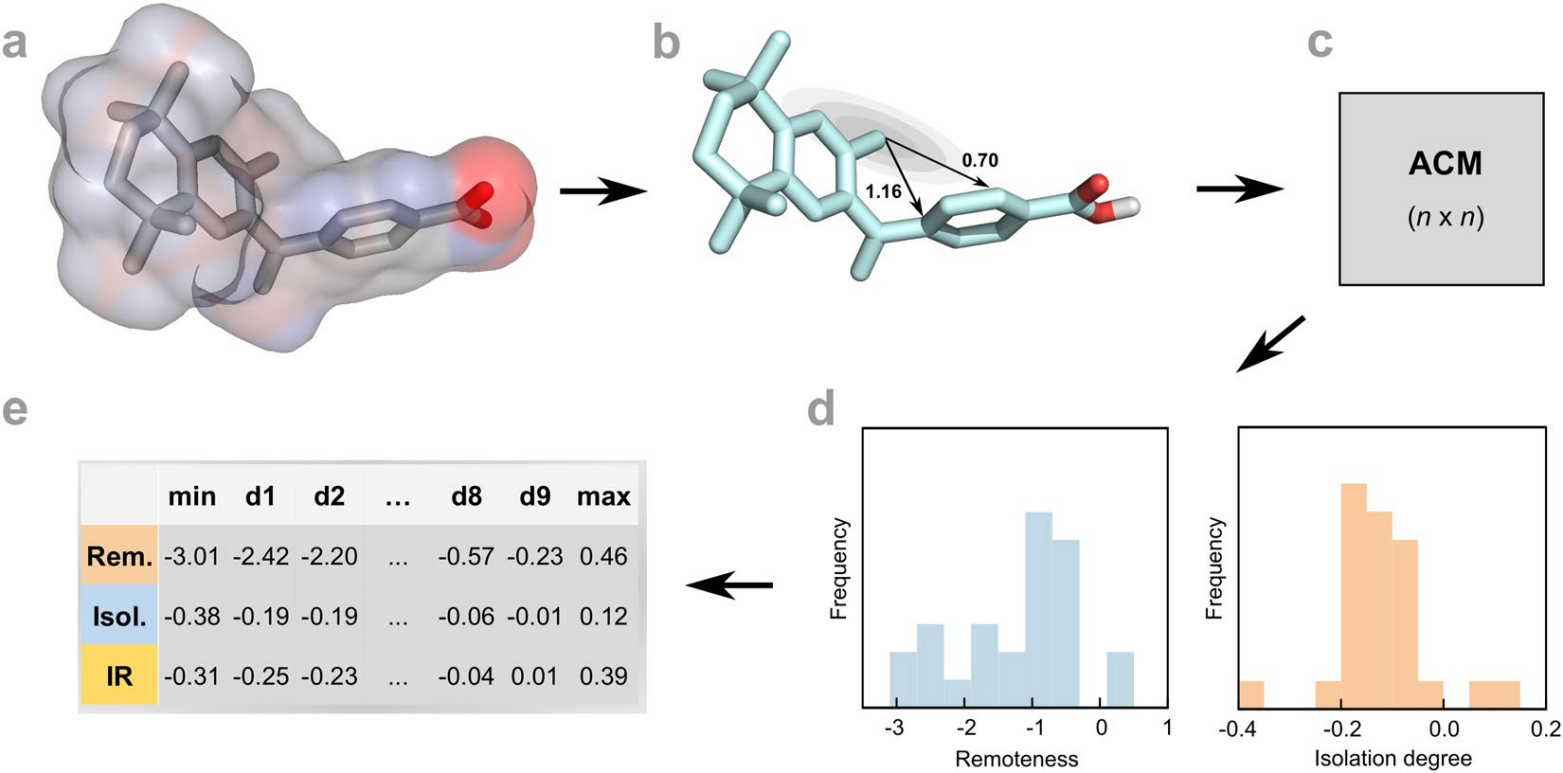
Simplified representation of WHALES calculation, taking the example of bexarotene. (**a**) Input chemical information for WHALES calculation, *i.e*., three-dimensional coordinates and partial charges. (**b**) Computed atom-centred interatomic distances for two pairs of atoms. The distances are normalized according to the atom-centred covariance (here depicted as an ellipsoid whose main axes are the directions of maximum variance), computed by considering the distribution of atoms and charges in the three-dimensional space (see Eq. 1). (**c**) Atom-centred covariance matrix (**ACM**), containing all the pairwise distances computed from each atomic centre (column) to each other atom (row). Only non-hydrogen atoms are considered. (**d**) Frequency distribution of remoteness (Rem) and isolation degree (Is) of the molecule, computed as row average and column minimum (diagonal elements excluded) of the ACM, respectively. Negatively charged atoms are assigned a negative sign of remoteness and isolation degree. (**e**) WHALES descriptors, computed as deciles (from d1 to d9, plus minimum and maximum) of remoteness, isolation degree and their ratio (IR), obtaining in total 33 molecular-size-independent descriptors (WHALES).
- *Step 2*. Calculation of the atom centred covariance-matrix for each non-hydrogen atom (Fig. 1b):

WHALES descriptors encode information on geometric interatomic distances, molecular shape and atomic properties in a holistic way^23^. Partial charges and atom distributions are captured by weighted locally-centred atom distances, computed for each atom position in a three-dimensional (3D) molecular conformation. The WHALES calculation procedure is performed in five steps:

- *Step 1*. Calculation of partial charges and retrieval of 3D conformations (Fig. 1a);

1 $${{\bf{S}}}_{w(j)}=\frac{{\sum }_{i=1}^{n}\,|{\delta }_{i}|\cdot ({{\bf{x}}}_{i}-{{\bf{x}}}_{j}){({{\bf{x}}}_{i}-{{\bf{x}}}_{j})}^{{\rm{T}}}}{{\sum }_{i=1}^{n}\,|{\delta }_{i}|},$$

where (**x**_*i*_ − **x**_*j*_) are the differences between the 3D coordinates of the *j*-th atomic centre and those of any *i*-th non-H atom; |δ_i_| is the absolute value of the partial charge of the *i*-th atom. The weighted covariance matrix (**S**_w(j)_) captures the distribution of atoms and their partial charges around each *j*-th atom.

- *Step 3*. Atom-Centred Mahalanobis distance (**ACM**) is computed as (Fig. 1c):
  2 $${\bf{A}}{\bf{C}}{\bf{M}}\,(i,j)={({{\bf{x}}}_{i}-{{\bf{x}}}_{j})}^{{\rm{T}}}\cdot {{\bf{S}}}_{w(j)}^{-1}\cdot ({{\bf{x}}}_{i}-{{\bf{x}}}_{j}).$$
  The **ACM** distance matrix collects all the pairwise normalized interatomic distances according to the atom-centred covariance matrix (Fig. 1b). Each *i*-th row represents the distance of the *i-*th atom from each atomic centre, whilst each *j*-th column contains the distances from atom *j* to all of the other atoms, where *j* itself is the centre of the molecular feature space. Atoms located in the directions of high variance will have a smaller distance from the *j-*th atomic centre than atoms located in low-variance regions, *e.g*., peripheral and sparsely populated regions.
- *Step 4*. Calculation of atomic parameters. From the **ACM** matrix (diagonal elements excluded), the remoteness and isolation degrees are computed as the row average and the column minimum, respectively. Additionally, the ratios of isolation degree to remoteness value are computed. Negatively-charged atoms are assigned a negative value of isolation degree, remoteness and their ratio (Fig. 1d);
- *Step 5*. Calculation of molecular descriptor vectors. To produce descriptors independent of molecular size, the distribution of atomic remoteness, isolation degree and the ratio of these is captured by calculating minimum, maximum and decile values. These 33 values constitute the WHALES descriptors (Fig. 1e).

In this present work, MMFF94^25^ energy-minimized structures were used for WHALES calculations. Two methods for the calculation of partial charge were employed for comparison, as explained in the next section.

### Benchmark analysis

WHALES descriptors were tested for their scaffold-hopping potential in three versions, with decreasing levels of complexity according to the partial charge specification (δ_i_, Eq. 1):

1. *WHALES-DFTB*+, computed by utilizing DFTB+^26^ for partial charge calculation, which is based on the density-functional-based tight-binding (DFTB) approach, providing an accelerated quantum mechanical simulation of partial charge, by making use of several approximations tailored for small molecules.
2. *WHALES-GM*, which utilizes the Gasteiger-Marsili^27^ method, developed for rapid calculations of partial charges according to the atom connectivity;
3. *WHALES-shape*, in which no information about the charge is used (*i.e*., δ_i_ = 1 for all atoms, Eq. 1) and only the atomic 3D coordinates are utilized.

These three versions represent distinct levels of the atomistic detail included in each representation, from the most chemically-detailed (WHALES-DFTB+) to the most abstract (WHALES-shape), where only the atom positioning is considered.

To benchmark the scaffold-hopping ability of WHALES, we chose seven state-of-the-art molecular descriptors, selected to cover different molecular “dimensionalities” (0D to 3D descriptors), and domains of encoded chemical information:

1. *Constitutional descriptors* (“Const”, 0D/1D)^28^, which capture basic structural properties of chemicals, such as molecular weight, number and percentage of carbon atoms, rings and heteroatoms.
2. *MACCS 166 keys* (“MACCS”, 1D)^8^, based on the presence of 166 predefined substructures;
3. *Extended Connectivity Fingerprints* (“ECFPs”, 1D)^7^, which are based on the presence of atom-centred radial fragments;
4. *Chemically Advanced Template Search 2* (“CATS”, 2D)^9^, based on the scaled occurrence of pharmacophore feature pairs (lipophilic, aromatic, hydrogen-bond acceptor, hydrogen-bond donor atoms) at a given topological distance;
5. *Matrix-based descriptors* (“MB”, 2D)^11,28^, which are based on graph theory and capture information regarding molecular branching, shape, saturation and the presence of heteroatoms;
6. *Weighted Holistic Invariant Molecular descriptors* (“WHIM”, 3D)^29^, which capture 3D information on the distribution of atoms and molecular properties (molecular mass, van-der-Waals volume, electronegativity, polarizability, ionization potential, intrinsic state) along principal molecular axes.
7. *GEometry Topology and Atom-Weights AssemblY* (“GETAWAY”, 3D)^30^, which account for the size and shape of the molecule, atom types, bond multiplicity and atomic properties (molecular mass, van-der-Waals volume, atom electronegativity, atom polarizability, ionization potential and intrinsic state), by calculating a weighted leverage value on the atomic coordinates.

To assess the potential of WHALES for scaffold-hopping compared with the benchmarks, we performed a retrospective virtual screening on 30,000 bioactive compounds (IC/EC_50_, K_d_/K_i_ values < 1 μM) extracted from the ChEMBL22^24^ compound database. For each biological target with at least 20 annotated actives (*n* = 182), each active was used in turn as the query to perform a similarity search. In analogy to a recent study^9^, the scaffold-hopping ability of each descriptor was calculated as the relative scaffold diversity of actives in the top 5% (SD_A_%) of each ranked list, defined as follows (Eq. 3):

3 $$S{D}_{A} \% =\frac{ns}{na}\cdot 100,$$

where *ns* is the number of unique Murcko^31^ scaffolds identified in the top 5% molecules of the ranked list, while *na* is the number of actives present in that same portion of the ranking. In other words, SD_A_% is the ratio of scaffolds (*ns*) to the number of retrieved actives (*na*) in the top 5% portion of the respective screening runs.

**Figure 2:**
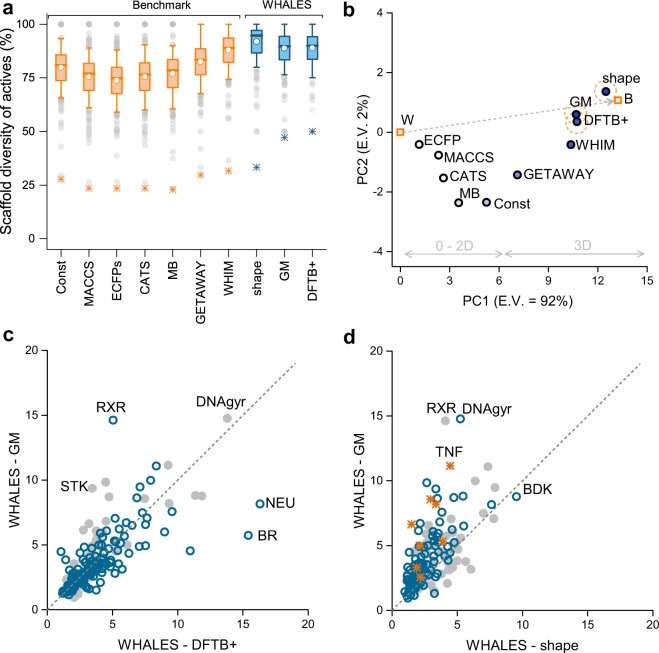
Retrospective virtual screening on known bioactives. 30,000 ChEMBL bioactive compounds (IC/EC_50_, K_d_, K_i_ values < 1 μM) on 182 biological targets were used for virtual screening with three versions of WHALES (GM, DFTB+, shape) and seven state-of-the-art molecular descriptors. (**a**) Relative scaffold diversity of actives for each descriptor on each dataset, expressed as the ratio of differing scaffolds to the number of retrieved actives among the top 5% portion of the respective screening runs. Boxplots show the median (line), mean (white dot), standard deviation (box edges), 5^th^ and 95^th^ percentiles (whiskers); grey dots represent outliers; asterisks denote the minimum value. WHALES descriptors produced a significantly higher relative scaffold diversity of actives (*p* < 0.01, Kruskal-Wallis^32^ with Dunn’s post-hoc analysis^33^), except for WHALES-GM and WHALES-DFTB+ compared to WHIM (*p* = 1.00); (**b**) Principal Component Analysis (PCA) performed on the SD_A_% values obtained by each descriptor on each biological target (first two PCs depicted, E.V. = explained variance). B and W denote the highest and lowest value produced by the pool of descriptors on each biological receptor; the dashed line represents the variation from the worst to the best relative scaffold diversity on average. Descriptors (circles) are coloured according to their mean SD_A_%, from white (low) to blue (high). WHALES descriptors (dashed circle) have the largest SD_A_% on average. (**c**) Comparison between the enrichment factor (EF_1%_) of WHALES-GM and WHALES-DFTB+. Blue dots represent the cases where the SD_A_% of WHALES-GM in the top 1% of the list was more than 3% larger than WHALES-DFTB+. In no case the SD_A_% of WHALES -DFTB+was more than 3% larger than that of WHALES-GM. (**d**) Comparison between the enrichment factor (EF_1%_) of WHALES-GM and WHALES-shape. Blue dots represent the cases where the SD_A_% of WHALES-GM in the top 1% of the list was more than 3% larger than WHALES-shape; the opposite case is represented by orange asterisks; grey circles denote biological targets with similar SD_A_%. Molecular targets for which WHALES performed well in terms of enrichment are highlighted in (**c**) and (**d**) with the following labels: BDK = bradykinin receptor, BR = bombesin receptor, DNAgyr = DNA gyrase, NEU = neuraminidase, RXR = retinoid X receptor, STK = serine/threonine protein kinase (PIKK family).

All of the analysed descriptors showed satisfactory scaffold-hopping ability in this benchmark study (Fig. 2a), with the lowest values observed for fingerprint-based representations, *i.e*., ECFPs (SD_A_% = 73 ± 12) and MACCS FP (SD_A_% = 75 ± 12), which rely on the presence of molecular fragments. The three versions of WHALES descriptors showed the highest average scaffold-hopping ability, equal to SD_A_% = 92 ± 11, SD_A_% = 89 ± 11 and SD_A_% = 89 ± 11, for WHALES-shape, WHALES-GM and WHALES-DFTB+, respectively. Except for WHIM (average SD_A_% = 88 ± 11), the WHALES descriptors showed a significantly higher SD_A_% compared to the tested benchmark descriptors (*p* < 0.001, Kruskal-Wallis with post-hoc Dunn’s tests^32,33^).

To better evaluate the scaffold-hopping ability of the methods, a Principal Component Analysis (PCA)^34^ was performed on the obtained SD_A_% values. PCA is a multivariate statistical technique for data visualization and dimensionality reduction that linearly combines the original variables into new orthogonal variables (principal components [PCs]), such that the first PC explains the largest data variance, the second one (orthogonal to the first) the second largest variance, and so on. Thereby, one can analyse the linear relationships among the original data and the PCs. A matrix was constructed with as many rows as the number of analysed descriptors [*n* = 10] and as many columns as the SD_A_% obtained on each biological target [*p* = 182]. To enhance the interpretability of the PCA, two rows were added that contained the highest and lowest values of SD_A_% obtained on each biological target (named “Best” [B] and “Worst” [W], respectively). This additional information stretches the PCA results along the worst-best (W-B) direction, thereby allowing one to more easily identify the methods with better/worse performance on average. The deviation from the W-B direction gives an indication of the variability of the methods according to the analysed target. The first two components (PC1 and PC2) explain 94% of the total variance (Fig. 2b). The variation from the worst to the best descriptors (W-B line) is primarily explained by PC1 and relates to the scaffold hopping ability of the analysed methods (the higher the descriptor’s closeness to B in that direction, the higher the average SD_A_% on the 182 analysed biological targets) (Fig. 2b). Descriptors located on the right of the plot have a larger SD_A_% on average than descriptors located on the left, with PC1 clearly separating 0D, 1D and 2D molecular descriptors from 3D approaches, the latter having a higher scaffold-hopping ability on average. The three version of WHALES have the largest PC1 scores (in accordance with their highest scaffold-hopping ability on average), with the maximum value for WHALES-shape, followed by WHALES-GM and WHALES-DFTB+. The deviation from the W-B line increases when the scaffold-hopping variability varies for different molecular targets. Descriptors located close to the W-B line have a stable performance on all the biological targets considered, while descriptors far from this line perform differently on the targets analysed. The PCA space shows that WHALES-shape and WHALES-GM have the best compromise between scaffold-hopping ability and stability, as they lie close to the B-W line, and have the largest average SD_A_%. WHIM descriptors had a slightly lower SD_A_% than WHALES (Fig. 2a), and their scaffold-hopping ability appears to be more dependent on the chosen biological target (Fig. 2b).

When comparing the enrichment ability of the three sets of WHALES descriptors, similar performances were obtained by WHALES-GM and WHALES-DFTB+ (average Enrichment Factor [EF_1%_] equal to EF_1%_ = 3.9 ± 2.5 and EF_1%_ = 3.9 ± 2.8, respectively), while the shape-based version only led to EF_1%_ = 2.8 ± 1.5. The correlations between EF_1%_ for WHALES-GB and WHALES-DFTB+ (ρ = 0.73) highlight a small influence on the partial charge calculation method utilized for WHALES, as the molecular descriptors rely on partial charge differences rather than on the precise values, with WHALES-GM appearing more suited for retrieving bioactive molecules with relatively few heteroatoms (Supplementary Fig. 1). On the contrary, WHALES-shape have a lower correlation with WHALES-GB and WHALES-DFTB+, with ρ = 0.68 and ρ = 0.65, respectively. Based on the retrospective results, the Gasteiger-Marsili based WHALES produced the best compromise between scaffold-hopping ability, enrichment and computational cost.

### Prospective validation

To experimentally validate the scaffold-hopping ability of WHALES with Gasteiger-Marsili partial charges, we chose the retinoid X receptor (RXR) as a target of interest. On RXR, WHALES-GM showed desirable scaffold-hopping ability (SD_A_% = 79%), with, in addition, increased enrichment as compared to WHALES-DFTB+ and WHALES-shape (Fig. 2). RXRs play a key role in cell proliferation and differentiation, metabolic balance, inflammation, and cancer, and are obligate heterodimer partners for several other nuclear receptors^24^. Drugs that target RXR and its heterodimerization partners are employed in the clinic for the treatment and alleviation of cancer, dermatologic diseases, endocrine disorders and metabolic syndrome^35,36^. The known binders of RXR have a limited chemotype diversity: 90% of RXR actives annotated in ChEMBL (EC/IC_50_ < 50μM) contain only seven types of reduced graph scaffolds^37^. The clinical importance, and limited structural diversity, of this class of compounds advocate for the application of methods which facilitate scaffold-hopping from known RXR modulators into new chemical space.

**Figure 3:**
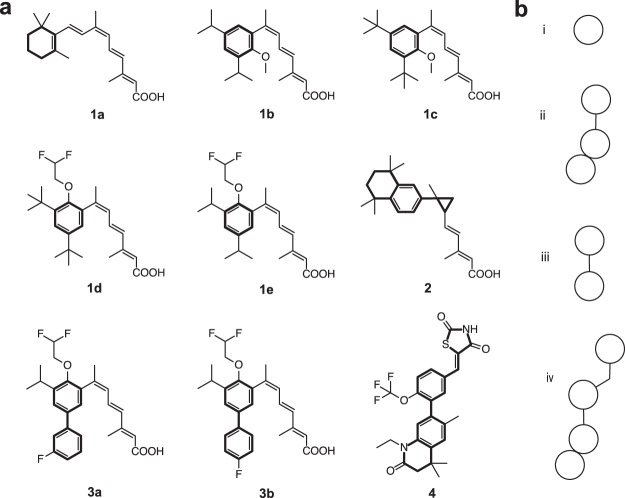
Queries utilized for the WHALES-GM-based virtual screening on commercially available compounds. (**a**) Query structures, labelled according to the scaffold type (from **1** to **4**), with Murcko^31^ scaffolds highlighted. (**b**) reduced scaffolds of the queries labelled with roman numerals (from i to iv). The reduced scaffolds i, ii and iii characterize 22%, 13% and 3% of the RXR actives annotated in ChEMBL23 (EC_50_/IC_50_ < 50 μM), respectively^37^.

The nine most potent binders according to K_i_, K_d_, and EC/IC_50_ as annotated in ChEMBL23 (Fig. 3, EC/IC_50_, K_i_/K_d_ < 0.8 μM) were chosen as queries for the prospective application. The scaffold diversity of these queries is limited, as only four scaffolds (**1**–**4**, Fig. 3) are present. Each active query was used in turn to perform an independent similarity-based virtual screening on a library containing 3,383,942 commercially available synthetic compounds. The Euclidean distance calculated on Gaussian-normalized WHALES between each query and the library compounds was used as a ranking criterion. Compounds were then sorted according to the sum of their reciprocal ranks obtained with each query, which is known to increase the enrichment ability of virtual screening protocols compared to using a single query^38^.

The 20 top-ranked synthetic compounds were selected and tested *in vitro* for their modulatory activity on RXR (Supplementary Table 1). Compounds were tested in specific hybrid reporter gene assays for RXRα, RXRβ and RXRγ modulation^39–41^. These assays rely on a constitutively expressed hybrid receptor composed of the respective human RXR ligand binding domain and the DNA binding domain of the Gal4 receptor from yeast. A Gal4 responsive firefly luciferase was used as reporter gene and constitutively expressed *renilla* luciferase served as internal control for transfection efficiency and test compound toxicity. All selected compounds were tested at 50 µM concentration on RXRα and for active compounds (Supplementary Fig. 1), dose response curves were recorded on all three RXR subtypes. Compounds **5**–**8** displayed partial RXR agonistic potency with intermediate micromolar EC_50_ values (EC_50_ values between EC_50_ = 14.7 ± 0.8 μM and EC_50_ = 32.1 ± 0.9; Table 1), without pronounced subtype preference.

**Table 1:** *In vitro* activity of the hits identified by WHALES-GM on RXRα/β/γ. EC_50_ ± SEM [µM] is reported (n ≥ 4).

ID	Compound	RXRα [µM]	RXRβ [µM]	RXRγ [µM]
**5**		17 ± 1	14.7 ± 0.8	15.0 ± 0.6
**6**		16.5 ± 0.6	16.6 ± 0.2	16 ± 4
**7**		32.1 ± 0.9	25.1 ± 0.1	28.2 ± 0.1
**8**		23.7 ± 0.7	25.6 ± 0.4	32 ± 3

**Figure 4:**
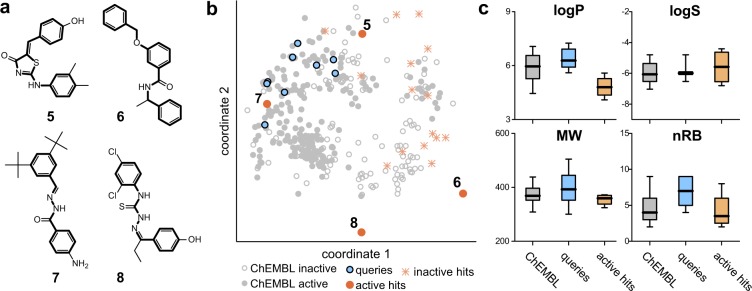
Analysis of the hits obtained with WHALES-GM on RXR receptors. (**a**) Scaffolds of the active hits identified by WHALES–GM (**5**–**8**, bold, cf. Table 1). None of these scaffolds was present in the ChEMBL23 annotated modulators. (**b**) Fragment analysis of hits and queries compared with known ChEMBL agonists (EC_50_ < 50 μM) and inactives (EC_50_, IC_50_, K_i_, K_d_ > 50 μM) on RXR. A multi-dimensional scaling (MDS) was performed on the extended connectivity fingerprints (1024-bit, radius = 0 to 3 bonds, 2 bits per pattern). Colours represent the set considered (grey = active and inactive compounds from ChEMBL, blue = queries, orange = WHALES hits); active hits are labelled with their ID (cf. Table 1). (**c**) Lead-likeliness of ChEMBL agonists, queries and active hits evaluated according to octanol-water partitioning coefficient (SlogP), solubility (AlogS), molecular weight (MW) and number of rotatable bonds (nRB)^43^.

**Figure 5:**
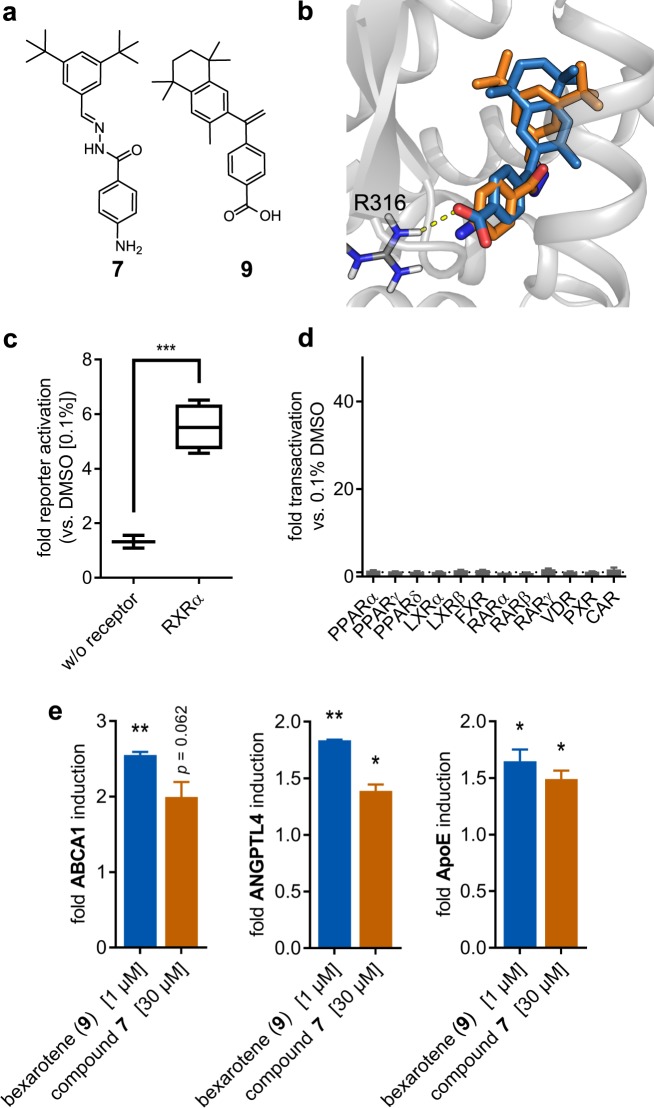
*In silico* and *in vitro* analysis of hit **7**. (**a**) Drug approved RXR agonist bexarotene (**9**), which was used as the reference for the analysis; (**b**) Comparison between the predicted binding poses of **7** (orange) and bexarotene (blue) in the ligand binding site of RXRα. The crystal structure of RXRα in complex with the agonist 9cUAB30 and the coactivator peptide GRIP-1 (PDB-ID: 4K4J) was prepared in MOE (v2016.0802)^57^, following the default protein preparation protocol. Structure energy was minimized using Amber10:EHT force field. For each ligand (*i.e*., crystalized ligand, bexarotene and hit **7**) 60 poses were generated, their energy was minimized using MMFF94x force field within a rigid receptor, and they were ranked by London dG score^57^; the top 10 poses were refined and scored using GBVI/WSA dG^57^ and the top-scoring pose was chosen. **7** and bexarotene share a similar binding pose, with **7** missing the interaction with R316 due to its lack of an acidic feature. (**c**) Control experiment: In absence of a Gal4-RXR hybrid receptor, the Gal4-responsive reporter gene was not transactivated by **7** confirming RXR-mediated activity. (**d**) RXR ligand **7** is highly selective over twelve related nuclear receptors (peroxisome proliferator-activated receptor [PPARα/γ/δ], liver X receptor [LXRα/β], farnesoid X receptor [FXR], retinoic acid receptor [RARα/β/γ], Vitamin D Receptor [VDR], pregnane X receptor [PXR], constitutive androstane receptor [CAR]). (**e**) RXR modulator **7** induces RXR regulated genes ATP-binding cassette transporter A1 (ABCA1), angiopoietin like protein 4 (ANGPTL4) and Apolipoprotein E (ApoE) with an efficacy comparable to RXR agonist bexarotene.

The novel active hits possess different scaffolds (Fig. 4a) compared to the utilized queries. Additionally, none of the hits possesses a scaffold known in ChEMBL23 for RXR binders (EC/IC_50_ and K_i/D_ < 50 μM), nor is annotated in the patent database SureChEMBL (Q1 2017)^42^. Apparently, most of the WHALES hits populate uncharted regions of the chemical space compared to known ChEMBL23 RXR modulators (Fig. 4b). This observation is most prominent for the active hits **6** and **8**, which lie far from the bulk on compounds annotated for RXR activity in ChEMBL. Both the active and inactive hits have a homogeneous distribution in the ChEMBL chemical space in terms of atom-centred fragments (as encoded by ECFPs), thus confirming the “fuzzy” nature of WHALES (Fig. 4b). Moreover, the identified active hits possess some desirable lead-like features (Fig. 4c)^43^, showing preferable lipophilicity, solubility, molecular weight and number of rotatable bonds compared to the utilized queries. Additionally, **6** and **7** are non-acidic RXR agonists (predicted pKa = 12.80 and pKa = 12.82, respectively), which is a rare feature amongst known RXR ligands^44^ (queries’ predicted pKa ranging from pKa = 4.17 to pKa = 6.35, Supplementary Table 2). **7** has a similar predicted binding pose to bexarotene (**9**, Fig. 5a), suggesting that WHALES descriptors capture relevant features for compound positioning in the active pocket. For these characteristics, we selected **7** for a broader *in vitro* characterization.

Control experiments not involving a hybrid receptor but only the reporter gene construct and the *renilla* luciferase showed no reporter transactivation confirming RXR mediated activity of **7** (Fig. 5b). Selectivity profiling on twelve related nuclear receptors (peroxisome proliferator-activated receptor [PPARα/γ/δ], liver X receptor [LXRα/β], farnesoid X receptor [FXR], retinoic acid receptor [RARα/β/γ], vitamin D receptor [VDR], pregnane X receptor [PXR], constitutive androstane receptor [CAR]) revealed no activity of **7** (Fig. 5c) and **7** showed no cytotoxic effect up to 100 µM (Fig. 5d). Moreover, **7** was characterized for its ability to modulate RXR target gene expression under more physiological conditions. Hepatoma cells (HepG2) were incubated with **7** (30 µM) or reference RXR agonist bexarotene (**9**, 1 µM) for eight hours and mRNA expression of RXR regulated genes ATP-binding cassette transporter A1 (ABCA1), angiopoietin like protein 4 (ANGPTL4) and apolipoprotein E (ApoE) was analysed by quantitative real-time PCR. Compound **7** induced all three studied genes with comparable efficacy as RXR agonist bexarotene (Fig. 5e).

### Concluding remarks and perspectives

In this study, WHALES descriptors confirmed their scaffold-hopping ability for synthetic molecules, by outperforming seven state-of-the-art molecular descriptors. Apparently, 3D representations, such as WHALES, increase the scaffold diversity compared to 0D–2D molecular representations (*e.g*., binary fingerprints), with the Gasteiger-Marsili charge specification utilized in WHALES constituting a suitable level of chemical abstraction for scaffold hopping. In the prospective setting, the four newly identified RXR agonists comprising new scaffolds, plus a novel non-acidic active chemotype, ultimately validate the usage of WHALES for virtual screening of synthetic compounds. WHALES-based hits have desirable features for drug design, as they possess novel chemotypes and improved lead-likeness compared to the queries. The level of abstraction from the molecular scaffolds obtained with WHALES makes these descriptors suitable for advancing medicinal chemistry projects, by allowing the exploration of uncharted regions in the chemical space. The possibility to include any desired atomic property as WHALES weighting scheme in addition to partial charges (Eq. 1) makes the method suitable for further tuning on a case-by-case basis, thereby bearing promise for innovative applications in drug discovery and chemical biology.

## Methods

### Molecule pre-processing

Molecule sanitization was performed using the tools made available in the RDKit^45^ (v. 2015.09.2) for checking and adjusting the valence, annotated aromaticity, conjugation and hybridization on a per-atom and per-bond basis for each molecule (“SanitizeMol” for molecule sanitization; “MolFromSmarts” to neutralise functional groups, correct errors in representation of aromatic nitrogen). Salts and counter ions were removed. We employed the MMFF94^25^ force field with 1000 iterations and 10 starting conformers for each compound (“EmbedMultipleConfs” [pruneRmsThresh = True, useBasicKnowledge = True, useExpTorsionAnglePrefs = True, useRandomCoords = True, numConfs = 10] and “MMFFOptimizeMoleculeConfs” [mmffVariant = ‘mmff94’, maxIters = 1000]); the lowest-energy conformer for each molecule was used for the subsequent 3D descriptor calculation.

### Charge calculation

(a) Gasteiger-Marsili^27^ partial charges were computed using RDKit^45^ v. 2015.09.2 and default settings. (b) DFTB+ partial charges were calculated with DFTB+^26^ (v. 1.2.2), with Slater-Koster^46^ tight-binding “mio” and “3ob” sets, extended with the “mio:hh” and “mio:nh” subsets, to improve the accuracy of nitrogen-hydrogen energy assessments. Hubbard^47^ derivatives were chosen according to default parameters suggested in the documentation. Angular momentum was limited in accordance with default parameters. Hydrogen-X damping was enabled, with an exponent of 4. Electronic temperature was 300 K. Drivers were disabled, as we wished to describe the energetics of our minimised structures. The SCC-DFTB Hamiltonian was used for the calculations, which were carried out with the Relatively Robust Hamiltonian Eigensolver^48^, with an operational tolerance value of 10^−5^ for convergence, and a maximum of 100 iterations. A failure to reach convergence in 100 iterations results in the repetition of the simulation, with an upper limit of 1000 iterations. Molecules which did not reach SCC after the 1000-iteration cycle were discarded, as were those where we lacked parameter sets for each of their atoms.

### Descriptors calculation

WHALES descriptors were calculated with in-house software written in python and available at as an open source GitHub repository (https://github.com/grisoniFr/whales_descriptors.git). MACCS166 keys were computed with RDKit module with default settings; all the other descriptors were calculated with Dragon 7^49^ (ECFP settings: size = 1024 bit; 2 bit per pattern, length = 0 to 2 bonds; count fragments = true, atom options = [Atom type, Aromaticity, total connectivity, charge, bond order]).

### Retrospective screening

A set of 469,123 active compounds annotated for their activity against 1,013 targets was collected from CheMBL22 database^50,51^. Disconnected structures and salts were removed and a set of 30,000 compounds was randomly extracted with a stratified resampling, *i.e*., by preserving the proportion of the actives for each target. For each target subtype with more than 20 annotated ligands (182 targets), each active was used as a query in turn to retrieve all the other ones on the basis of similarity calculated on WHALES and benchmark descriptors. For the real-valued descriptors, the Euclidean distance on Gaussian-normalized data was utilized, while for binary descriptors, the Jaccard-Tanimoto similarity coefficient was utilized^11^. Scaffold diversity was calculated considering Murcko scaffolds^31^ computed with RDKit. For each biological target, the SD_A_% was calculated of the median of the values retrieved by each retrospective run.

### Commercial compound library

The library was assembled from commercially available synthetic compounds from Asinex^52^ (Elite, Fragments, Gold & Platinum collections), ChemBridge screening compound collection^53^, Enamine advanced and HTS collections^54^, and Specs screening compounds^55^.

### Comparison with RXR agonists from ChEMBL

EC_50_, IC_50_, K_i_ and K_d_ data were downloaded from ChEMBL23 (human RXRα, RXRβ and RXRγ). Records whose data curation was labelled as of intermediate quality were removed. Records whose activity was labelled as “not determined” were removed. Compounds with EC_50_ ≤ 50 μM were considered as active. Compounds labelled as non-active or having EC_50_, IC_50_, K_i_, K_d_ > 50 μM were considered as inactive. Records were merged according to canonical SMILES and compounds with conflicting activity annotations were removed. Compounds were standardized with RDKit normalizer^45^; failed molecules were removed. The set of utilized ChEMBL compounds is provided as supporting material (Supplementary Table 3). Extended Connectivity Fingerprints (ECFP) were computed with Dragon 7^49^ (length = 1024 bit, radius = 0 to 3 bonds, bits per pattern = 2, count fragments = true, atom options = [Atom type, Aromaticity, total connectivity, charge, bond order]). The non-parametric multi-dimensional scaling was performed with MATLAB cmdscale function on the intermolecular Jaccard-Tanimoto distances (two coordinates, final stress error = 0.34). Molecular weight (MW), number of rotatable bonds and SlogP were calculated with RDKit^45^; AlogS was calculated with VCCLAB^56^. The pKa values of ChEMBL compounds, hits and queries were predicted with the ChemAxon Chemicalize module (https://chemicalize.com, accessed September 2018).

### Docking

The crystal structure of RXRα in complex with the agonist 9cUAB30 and the coactivator peptide GRIP-1 (PDB-ID: 4K4J) was prepared in MOE (v2016.0802)^57^, with the QuickPrep module (Structure Preparation = True; Protonate3D = True [T = 300, pH = 7; Salt = 0.1; Electrostatics = GB/VI; Cutoff = 15; Dielectric = 2; Solvent = 80; van der Waals = 800R3]; ASN/GLN/HIS flips allowed = True; protonation at pH = 7; correction of structural issues [missing residues and incorrect hybridization]; removal of water molecules farther than 4.5 Å from the receptor or ligand; restriction of receptor atoms positions [force constant = 10, buffer = 0.25 Å]; fixed position of all atoms farther away than 8 Å from the ligand). The protein structure was minimised using Amber10:EHT force field (termination value = 0.1 kcal × mol^−1^ × Å^−1^). Ligands were protonated at pH = 7; for each ligand (*i.e*., crystalized ligand, bexarotene and hit **7**) 60 poses were generated, their energy was minimized using MMFF94x force field within a rigid receptor, and they were ranked by London dG score^57^; the top 10 poses were refined and scored using GBVI/WSA dG^57^ and the top-scoring pose was chosen. Re-docking of the crystallized ligand following such protocol led to small RMSD values (final pose: 0.365 Å).

### Hybrid reporter gene assays for PPARα/γ/δ, LXRα/β, RXRα/β/γ, RARα/β/γ, FXR, VDR, CAR and PXR

The Gal4 hybrid reporter gene assays were conducted as reported previously^39–41^. pFA-CMV-based constructs comprising the ligand binding domain of the human nuclear receptor in question were used as expression plasmids for the chimera receptors. pFR-Luc (Stratagene) served as reporter plasmid and pRL-SV40 (Promega) for normalization of transfection efficiency and cell growth. The assays were conducted in 96-well format in HEK293T cells that were cultured as described previously^39–41^. Transient transfection was carried out using Lipofectamine LTX reagent (Invitrogen) according to the manufacturer’s protocol. After transfection and incubation with test compounds (12–14 h), cells were assayed for luciferase activity using Dual-Glo™ Luciferase Assay System (Promega) according to the manufacturer’s protocol. Luminescence was measured with an Infinite M200 luminometer (Tecan Deutschland GmbH). All hybrid assays were validated with reference agonists (PPARα: GW7647; PPARγ: pioglitazone; PPARδ: L165,041; LXRα/β: T0901317; FXR: GW4064; RXRs: bexarotene; RARs: tretinoin; VDR: calcitriol; CAR: CITCO; PXR: SR12813) which yielded EC_50_ values in agreement with literature. The assays were conducted in duplicates with at least two independent repeats and for active compounds repeated without hybrid receptor coding DNA for every test compound at the highest tested concentration to exclude unspecific effects.

### Target gene quantification (quantitative real-time PCR)

HepG2 cells were incubated with test compound **7** (30 µM) or bexarotene (1 µM) as positive control each dissolved in 0.1% DMSO or 0.1% DMSO alone as untreated control for 8 h, harvested, washed with cold phosphate buffered saline (PBS) and then directly used for RNA extraction with the Total RNA Mini Kit (R6834-02, Omega Bio-Tek, Inc., Norcross, GA, USA). 2 µg RNA were reverse-transcribed into cDNA using the High-Capacity cDNA Reverse Transcription Kit (4368814, Thermo Fischer Scientific, Inc.). RXR target gene expression was evaluated by quantitative real time PCR analysis with a StepOnePlus™ System (Life Technologies, Carlsbad, CA, USA) using PowerSYBRGreen (Life Technologies; 12.5 µl per well) and the primers reported in Supplementary Table 4^58^. Each sample was set up in duplicates and repeated in two independent experiments. The expression was quantified by the comparative ∆∆Ct method. Glycerinaldehyde 3-phosphate dehydrogenase (GAPDH) was used as reference. Results are expressed as mean ± standard error of the mean (SEM) relative mRNA expression compared to DMSO (0.1%) control which was set as 1.

### Toxicity assay (water-soluble tetrazolium assay)

WST-1 assay (Roche Diagnostics International AG, Rotkreuz, Schweiz) was performed according to manufacturer’s protocol in HepG2 cells. Cells were incubated with the test compounds (final concentrations: 1 µM, 10 µM, 30 µM, 50 µM and 100 µM) in DMEM/1% DMSO, and DMEM/1% DMSO as negative control. After 48 h, WST reagent (Roche Diagnostics International AG) was added to each well according to manufacturer’s instructions. After 45 min incubation, absorption (450 nm/reference: 620 nm) was determined with a Tecan Infinite M200 (Tecan Deutschland GmbH). Each experiment was set up in duplicates and repeated in four independent experiments. Results are expressed as mean ± SEM% of DMSO (0.1%) control.

## Electronic supplementary material

Supporting Information

## Data Availability

The Python code implementing WHALES descriptors is deposited as an open source repository on GitHub (https://github.com/grisoniFr/whales_descriptors.git).
