# Supplementary material for: Scaffold-Hopping from Synthetic Drugs by Holistic Molecular Representation
Source: Sci Rep. 2018 Nov 7;8:16469. doi: 10.1038/s41598-018-34677-0 (PMC6220272; doi:10.1038/s41598-018-34677-0)
Supplement: Supplementary file 1 — Supporting Information [file 41598_2018_34677_MOESM1_ESM.docx]

**Scaffold-Hopping from Synthetic Drugs by Holistic Molecular Representation**

Francesca Grisoni,^1,2^* Daniel Merk,^1^ Ryan Byrne,^1^ Gisbert Schneider^1^*

^1^ Swiss Federal Institute of Technology (ETH), Department of Chemistry and Applied Biosciences, Vladimir-Prelog-Weg 4, CH-8093 Zurich, Switzerland

^2^ Milano Chemometrics & QSAR Research Group, Dept. of Earth and Environmental Sciences, University of Milano-Bicocca, IT-20126 Milano, Italy

*Corresponding authors: francesca.grisoni@unimib.it, gisbert.schneider@pharma.ethz.ch

**Supplementary Table 1.** 20 top-scoring hits identified by WHALES-GM on RXRα/β/γ. Compounds are labelled according to their rank. For active compounds (EC_50_ ±SEM [µM] ≤ 50 µM, n ≥ 4), the manuscript ID is also reported.

| rank | ID | Compound | rank | ID | Compound |
| --- | --- | --- | --- | --- | --- |
| 1 | n.a. |  | 11 | n.a. |  |
| 2 | **5** |  | 12 | **7** |  |
| 3 | n.a. |  | 13 | n.a. |  |
| 4 | n.a. |  | 14 | n.a. |  |
| 5 | n.a. |  | 15 | n.a. |  |
| 6 | n.a. |  | 16 | n.a. |  |
| 7 | n.a. |  | 17 | n.a. |  |
| 8 | n.a. |  | 18 | **8** |  |
| 9 | **6** |  | 19 | n.a. |  |
| 10 | n.a. |  | 20 | n.a. |  |

**Supplementary Table 2.** List of pKa values of queries and active hits identified by WHALES screening (cf. Table 1). Values were computed with ChemAxon Chemicalize (https://chemicalize.com/#/batch, accessed September 12, 2018).

| **ID** | **Type** | **Most acidic pKa** |
| --- | --- | --- |
| **1a** | Query | 4.76 |
| **1b** | Query | 4.19 |
| **1c** | Query | 4.17 |
| **1d** | Query | 4.17 |
| **1e** | Query | 4.18 |
| **2** | Query | 4.60 |
| **3a** | Query | 4.25 |
| **3b** | Query | 4.25 |
| **4** | Query | 6.35 |
| **5** | Active hit | 8.89 |
| **6** | Active hit | 12.80 |
| **7** | Active hit | 12.82 |
| **8** | Active hit | 9.07 |


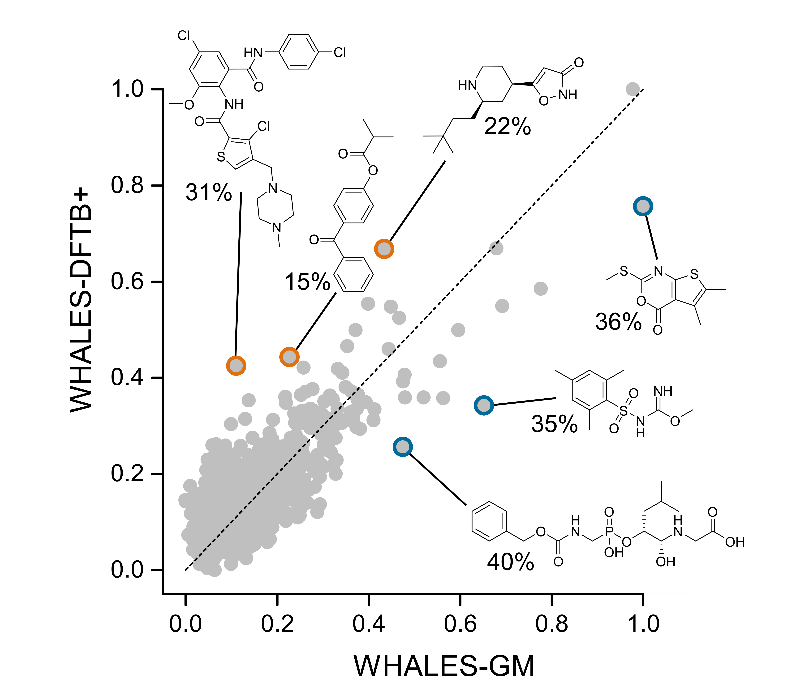


**Supplementary Figure 1.** Euclidean distances computed in one of the retrospective screening runs on Endopeptidase (Serine Endopeptidase, Serine Protease), using active compounds only (*n* = 942). Euclidean distances were range-scaled. The compounds with the highest differences between WHALES-GM and WHALES-DFTB+ distances are depicted. Numbers quantify the percentage of heteroatoms in each compound.


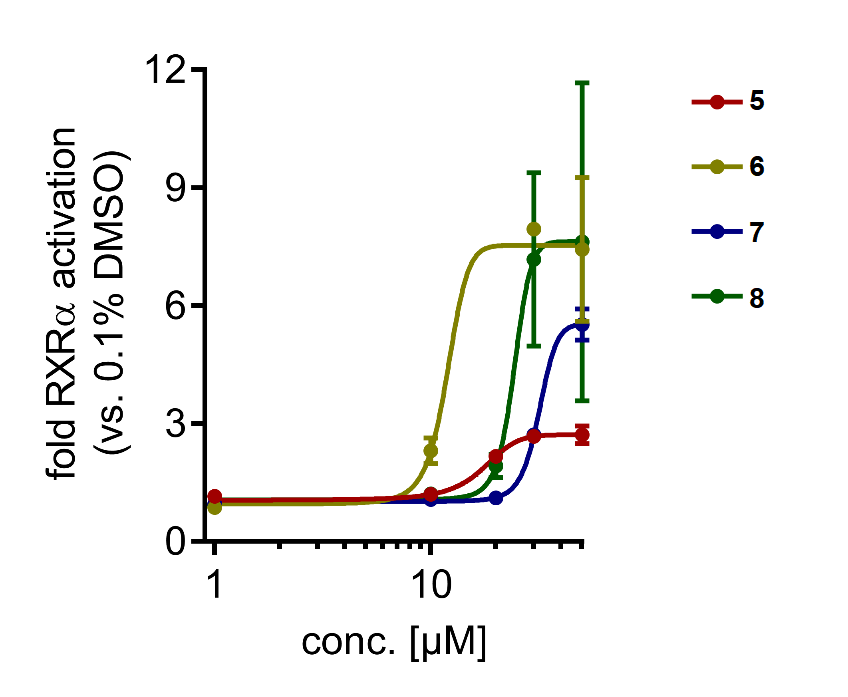


**Supplementary Figure 2.** Dose-response curves for RXRα activation. Compounds were tested in specific hybrid reporter gene assays for RXRα, RXRβ and RXRγ modulation.^1–3^ These assays rely on a constitutively expressed hybrid receptor composed of the respective human RXR ligand binding domain and the DNA binding domain of the Gal4 receptor from yeast. A Gal4 responsive firefly luciferase was used as reporter gene and constitutively expressed *renilla* luciferase served as internal control for transfection efficiency and test compound toxicity. All selected compounds were tested at 50 µM concentration on RXRα and for active compounds. Error bars represent the standard error of the mean (SEM).

**Supplementary Table 3.** SMILES of the utilized compounds in the present study (active and inactive compounds from ChEMBL, queries and WHALES hits).

| **Canonical SMILES (RDKit)** | **Type** |
| --- | --- |
| CC(C)=C(/C=C/C(C)=C/C(=O)O)c1cc2c(cc1C)C(C)(C)CCC2(C)C | active |
| CC(C)=C(/C=C/C(C)=C/C(=O)O)c1ccc2c(c1)C(C)(C)CCC2(C)C | active |
| CC(C)=C(c1ccc(C(=O)O)cc1)c1cc2c(cc1C)C(C)(C)CCC2(C)C | active |
| CC(C)=C(c1ccc(C(=O)O)cc1)c1ccc2c(c1)C(C)(C)CCC2(C)C | active |
| CC(=O)NCCCn1c2c(c3ccc(N)cc3c1=O)C(=O)c1ccccc1-2 | active |
| CC(=C\C=C\C(C)=C\C(=O)O)/C=C1\CCCC(C2CC2)=C1C(C)C | active |
| CC(=C\C=C\C(C)=C\C(=O)O)/C=C1\CCCC(c2ccccc2)=C1C(C)C | active |
| CC(=C\C=C\C(C)=C\C(=O)O)/C=C1\CCCC(C(C)C)=C1CCC(C)C | active |
| CC(=C\C=C\C(C)=C\C(=O)O)/C=C1\CCCC(C2CC2)=C1CCC(C)C | active |
| CC(=C\C=C\C(C)=C\C(=O)O)/C=C1\CCCC(c2ccccc2)=C1CCC(C)C | active |
| Cc1cc2c(cc1/C(=N\OCC(C)C)c1ccc(C(=O)O)cc1)C(C)(C)CCC2(C)C | active |
| CC(/C=C/CN(c1cc(C(C)(C)C)cc2c1CCC2(C)C)C(C)C)=C\C(=O)O | active |
| C/C(=C\C(=O)O)c1ccc2scc(-c3cc(C(C)C)cc(C(C)C)c3O)c2c1 | active |
| C/C(=C\C(=O)O)c1ccc2scc(-c3cc(C(C)C)cc(C(C)C)c3OCCCF)c2c1 | active |
| CC(/C=C/C=C(\C)c1cc(C(C)C)cc(C(C)C)c1OCCF)=C\C(=O)O | active |
| C=C(c1ccc(C(=O)O)cc1)c1cc2c(cc1C(C)C)C(C)(C)CCC2(C)C | active |
| CC(C)c1cc2c(cc1C(=O)c1ccc(C(=O)O)cc1)C(C)(C)CCC2(C)C | active |
| Cc1cc2c(cc1C(C)c1ccc(C(=O)O)cc1)C(C)(C)CCC2(C)C | active |
| CC(c1ccc(C(=O)O)cc1)c1ccc2c(c1)C(C)(C)CCC2(C)C | active |
| C=C(c1ccc(C(=O)O)cc1)c1cc2c(cc1Br)C(C)(C)CCC2(C)C | active |
| C=C(c1ccc(C(=O)O)cc1)c1cc2c(cc1Cl)C(C)(C)CCC2(C)C | active |
| C=C(c1ccc(C(=O)O)cc1)c1cc2c(cc1F)C(C)(C)CCC2(C)C | active |
| C=C(c1ccc(C(=O)O)cc1)c1cc2c(cc1O)C(C)(C)CCC2(C)C | active |
| CC1(C)CCC(C)(C)c2cc(C(=O)c3ccc(C(=O)O)cc3)c(Br)cc21 | active |
| CC1(C)CCC(C)(C)c2cc(C(=O)c3ccc(C(=O)O)cc3)c(Cl)cc21 | active |
| CC1(C)CCC(C)(C)c2cc(C(=O)c3ccc(C(=O)O)cc3)c(F)cc21 | active |
| CC1(C)CCC(C)(C)c2cc(Cc3ccc(C(=O)O)cc3)ccc21 | active |
| CC1(C)CCC(C)(C)c2cc(/C(=N/O)c3ccc(C(=O)O)cc3)c(Br)cc21 | active |
| C=C(c1ccc(C(=O)O)cc1)c1ccc2c(c1)C(C)(C)CCC2(C)C | active |
| CC1(C)CCC(C)(C)c2cc(C(=O)c3ccc(C(=O)O)cc3)ccc21 | active |
| CC1(C)CCC(C)(C)c2cc(C(O)c3ccc(C(=O)O)cc3)ccc21 | active |
| CC1(C)CCC(C)(C)c2cc(C3(c4ccc(C(=O)O)cc4)CC3)ccc21 | active |
| CC1(C)CCC(C)(C)c2cc(C3(c4ccc(C(=O)O)cc4)OCCO3)ccc21 | active |
| CC1(C)CCC(C)(C)c2cc(C3(c4ccc(C(=O)O)cc4)OCCS3)ccc21 | active |
| CC1(C)CCC(C)(C)c2cc(C3(c4ccc(C(=O)O)cc4)SCCCS3)ccc21 | active |
| CC1(C)CCC(C)(C)c2cc(C3(c4ccc(C(=O)O)cc4)SCCS3)ccc21 | active |
| CC1(C)CCC(C)(C)c2cc(C3CCCc4oc(/C=C5\SC(=O)NC5=O)cc43)ccc21 | active |
| CC1(C)CCC(C)(C)c2cc(C3CCCc4oc(/C=C/C(=O)O)cc43)ccc21 | active |
| CC1(C)CCC(C)(C)c2cc(C3CCCc4oc(/C=C/C(=O)O)nc43)ccc21 | active |
| CC1(C)CCC(C)(C)c2cc(C3CCCc4sc(CCC(=O)O)cc43)ccc21 | active |
| CC1(C)CCC(C)(C)c2cc(C3CCCc4sc(/C=C/C(=O)O)cc43)ccc21 | active |
| CC1(C)CCC(C)(C)c2cc(/C(=N/O)c3ccc(C(=O)O)cc3)ccc21 | active |
| CC1(C)CCC(C)(C)c2cc(-c3ccccc3-c3ccc(C(=O)O)cc3)ccc21 | active |
| CC1(C)CCC(C)(C)c2cc3c(cc21)OC1CCCC31c1ccc(C(=O)O)cc1 | active |
| CC1(C)CCC(C)(C)c2cc3c(cc21)OC1CCCCC31c1ccc(C(=O)O)c(F)c1 | active |
| CC1(C)CCC(C)(C)c2cc3c(cc21)OC1CCCCC31c1ccc(C(=O)O)cc1 | active |
| CC1=C(/C=C/C#Cc2ccc(C(=O)O)cc2)C(C)(C)CCC1 | active |
| C=C(c1ccc(C(=O)O)cc1)c1cc2c(cc1C)C(C)(C)C(C)C2(C)C | active |
| CCC(C)(C)c1cc(/C(C)=C/C=C/C(C)=C/C(=O)O)c(OC)c(C(C)(C)CC)c1 | active |
| CCC1=C(CCC(C)C)/C(=C/C(C)=C/C=C/C(C)=C/C(=O)O)CCC1 | active |
| CCCCCCOc1cc(N(CC)c2ccc(C(=O)O)cn2)ccc1C(C)C | active |
| CCCCCOc1cc(N(CC)c2ccc(C(=O)O)cn2)ccc1C(C)C | active |
| CCCCN(C/C=C/C(C)=C/C(=O)O)c1cc(C(C)(C)C)cc2c1CCC2(C)C | active |
| CCCCO/N=C(/c1ccc(C(=O)O)cc1)c1cc2c(cc1C)C(C)(C)CCC2(C)C | active |
| CCCCOc1c(/C(C)=C/C=C/C(=C/C(=O)O)C(F)(F)F)cc(C(C)(C)C)cc1C(C)(C)C | active |
| CCCCOc1c(/C(C)=C/C=C/C(C)=C/C(=O)O)cc(C(C)(C)C)cc1C(C)(C)C | active |
| CCCCOc1c(-c2c[nH]c3ccc(/C(C)=C/C(=O)O)cc23)cc(C(C)(C)C)cc1C(C)(C)C | active |
| CCCCOc1c(/C(C)=C/C=C/C(C)=C/C(=O)O)cc(C(C)C)cc1C(C)C | active |
| CCCCOc1c(-c2c[nH]c3ccc(/C(C)=C/C(=O)O)cc23)cc(C(C)C)cc1C(C)C | active |
| CCCCOc1c(-c2cccc3[nH]c(/C(C)=C/C(=O)O)cc23)cc(C(C)C)cc1C(C)C | active |
| CCCCOc1c(-c2cn(C)c3ccc(/C(C)=C/C(=O)O)cc23)cc(C(C)C)cc1C(C)C | active |
| CCCCOc1cc(N(CC)c2ccc(C(=O)O)cn2)ccc1C(C)C | active |
| CCCN(C(=O)/C=C/C(C)=C/C(=O)O)c1cc(C(C)(C)C)cc(C(C)(C)C)c1 | active |
| CCCN(C/C=C/C(C)=C/C(=O)O)c1cc(C(C)(C)C)cc(C(C)(C)C)c1 | active |
| CCCN(C/C=C/C(C)=C/C(=O)O)c1cc(C(C)(C)C)cc2c1CCC2(C)C | active |
| CCCN(C/C=C/C(C)=C/C(=O)O)c1cc2c(cc1C)C(C)(C)CCC2(C)C | active |
| CCCN(C/C=C/C(C)=C/C(=O)O)c1ccc2c(c1)C(C)(C)CCC2(C)C | active |
| CCCO/N=C(/c1ccc(C(=O)O)cc1)c1cc2c(cc1C)C(C)(C)CCC2(C)C | active |
| CCCOc1c(-c2c[nH]c3ccc(/C(C)=C/C(=O)O)cc23)cc(C(C)(C)C)cc1C(C)(C)C | active |
| CCCOc1cc(N(CC)c2ccc(C(=O)O)cn2)ccc1C(C)C | active |
| CCCOc1cc2c(cc1C1CCCc3oc(/C=C/C(=O)O)cc31)C(C)(C)CCC2(C)C | active |
| CCCOc1cc2c(cc1/C(C)=C/C=C/C(C)=C/C(=O)O)C(C)(C)CCC2(C)C | active |
| CCN(C/C=C/C(C)=C/C(=O)O)c1cc(C(C)(C)C)cc(C(C)(C)C)c1 | active |
| CCN(C/C=C/C(C)=C/C(=O)O)c1cc2c(cc1C)C(C)(C)CCC2(C)C | active |
| CCN(C/C=C/C(C)=C/C(=O)O)c1ccc2c(c1)C(C)(C)CCC2(C)C | active |
| CCN(c1ccc(C(C)C)c(OC(C)C)c1)c1ccc(C(=O)O)cn1 | active |
| C=C(C)COc1cc(N(CC)c2ccc(C(=O)O)cn2)ccc1C(C)C | active |
| CCN(c1ccc(C(C)C)c(OCC(C)C)c1)c1ccc(C(=O)O)cn1 | active |
| CCN(c1ccc(C(C)C)c(OCC(C)CF)c1)c1ccc(C(=O)O)cn1 | active |
| CCN(c1ccc(C(C)C)c(OCC(F)(F)F)c1)c1ccc(C(=O)O)cn1 | active |
| CCN(c1ccc(C(C)C)c(OCC2CC2)c1)c1ccc(C(=O)O)cn1 | active |
| CCN(c1ccc(C(C)C)c(OCC=C(C)C)c1)c1ccc(C(=O)O)cn1 | active |
| CCN(c1ccc(C(C)C)c(OCCCc2ccccc2)c1)c1ccc(C(=O)O)cn1 | active |
| CCN(c1ccc(C(C)C)c(OCCc2ccccc2)c1)c1ccc(C(=O)O)cn1 | active |
| CCN(c1ccc(C(C)C)c(OCc2ccccc2)c1)c1ccc(C(=O)O)cn1 | active |
| CCN(c1ccc(OCC(C)C)c(C(C)C)c1)c1ccc(C(=O)O)cn1 | active |
| CCN(c1ccc(OCC(C)CF)c(C(C)C)c1)c1ccc(C(=O)O)cn1 | active |
| CCN(c1ccc(C(=O)O)cn1)c1ccc2c(C(F)(F)F)cc(=O)n(C)c2n1 | active |
| CCN(c1ccc(C(=O)O)cn1)c1ccc2c(C(F)(F)F)cc(=O)n(CC(C)C)c2n1 | active |
| CCN(c1ccc(C(=O)O)cn1)c1ccc2c(C(F)(F)F)cc(=O)n(CC)c2n1 | active |
| CCN(c1ccc2c(c1)C(C)(C)CCC2(C)C)c1ccc(C(=O)O)cn1 | active |
| CCN(c1ccc2c(c1)C(C)(C)CCC2(C)C)c1ccc(-c2nnn[nH]2)cn1 | active |
| CCO/N=C(/c1ccc(C(=O)O)cc1)c1cc2c(cc1C)C(C)(C)CCC2(C)C | active |
| CCOc1c(/C(C)=C/C=C/C(C)=C/C(=O)O)cc(C(C)(C)C)cc1C(C)(C)C | active |
| CCOc1c(/C(=C/C=C/C(C)=C/C(=O)O)C(F)(F)F)cc(C(C)(C)C)cc1C(C)(C)C | active |
| CCOc1c(/C(C)=C/C=C/C(C)=C/C(=O)O)cc(C(C)C)cc1C(C)C | active |
| CCOc1c(/C(C)=C/C=C/C(C)=C/C(=O)O)cc(C(F)(F)C(F)(F)F)cc1C(C)C | active |
| CCOc1c(-c2[nH]nc3ccc(/C(C)=C/C(=O)O)cc23)cc(C(C)C)cc1C(C)C | active |
| CCOc1c(-c2cccc3cc(/C(C)=C/C(=O)O)oc23)cc(C(C)C)cc1C(C)C | active |
| CCOc1c(-c2cccc3cc(/C(C)=C/C(=O)O)sc23)cc(C(C)C)cc1C(C)C | active |
| CCOc1c(-c2cccc3sc(/C(C)=C/C(=O)O)cc23)cc(C(C)C)cc1C(C)C | active |
| CCOc1c(-c2csc3cnc(/C(C)=C/C(=O)O)cc23)cc(C(C)C)cc1C(C)C | active |
| CCOc1c(-c2noc3ccc(/C(C)=C/C(=O)O)cc23)cc(C(C)C)cc1C(C)C | active |
| CCOc1cc2c(cc1/C(C)=C/C=C\C(C)=C\C(=O)O)C(C)(C)CCC2(C)C | active |
| C=C(c1ccc(C(=O)O)cc1)c1cc2c(cc1CC)C(C)(C)CCC2(C)C | active |
| CCc1cc2c(cc1C(=O)c1ccc(C(=O)O)cc1)C(C)(C)CCC2(C)C | active |
| CCc1cc2c(cc1/C(C)=C/c1ccc(C(=O)O)cc1)C(C)(C)CCC2(C)C | active |
| CN(C)CCCn1c2c(c3ccc(Br)cc3c1=O)C(=O)c1ccccc1-2 | active |
| CN(C)CCCn1c2c(c3ccc(N)cc3c1=O)C(=O)c1ccccc1-2 | active |
| CN(C)CCCn1c2c(c3ccccc3c1=O)C(=O)c1ccccc1-2 | active |
| C/C(=C/c1ncc(C(=O)O)n1S(=O)(=O)N(C)C)c1cc2c(cc1C)C(C)(C)CCC2(C)C | active |
| CC(/C=C/CN(C)c1cc(C(C)(C)C)cc(C(C)(C)C)c1)=C\C(=O)O | active |
| CN1Cc2oc(/C=C/C(=O)O)cc2C(c2ccc3c(c2)C(C)(C)CCC3(C)C)C1 | active |
| CNCCCn1c2c(c3ccc(Br)cc3c1=O)C(=O)c1ccccc1-2 | active |
| CNCCCn1c2c(c3ccccc3c1=O)C(=O)c1ccccc1-2 | active |
| CNc1ccc2c3c(n(CCCN)c(=O)c2c1)-c1ccccc1C3=O | active |
| CO/N=C(/c1ccc(C(=O)O)cc1)c1cc2c(cc1C)C(C)(C)CCC2(C)C | active |
| CO/N=C(/c1ccc(C(=O)O)cc1)c1cc2c(cc1C)C(C)(C)CCC2(C)C | active |
| CO/N=C(/c1ccc(C(=O)O)cc1)c1ccc2c(c1)C(C)(C)CCC2(C)C | active |
| COc1c(/C(C)=C/C=C/C(C)=C/C(=O)O)cc(C(C)(C)C)cc1C(C)(C)C | active |
| COc1c(/C(C)=C/C=C/C(C)=C/C(=O)O)cc(C(C)C)cc1C(C)C | active |
| COc1c(-c2csc3ccc(/C(C)=C/C(=O)O)cc23)cc(C(C)C)cc1C(C)C | active |
| C=C(c1ccc(C(=O)O)cc1)c1cc2c(cc1OC)C(C)(C)CCC2(C)C | active |
| COc1cc2c(cc1C(=O)c1ccc(C(=O)O)cc1)C(C)(C)CCC2(C)C | active |
| COc1cc2c(cc1C1CCCc3oc(/C=C/C(=O)O)cc31)C(C)(C)CCC2(C)C | active |
| COc1cc2c(cc1/C(C)=C/C=C\C(C)=C\C(=O)O)C(C)(C)CCC2(C)C | active |
| COc1ccc2c(c1)C(=O)c1c-2n(CCCN)c(=O)c2ccccc12 | active |
| C/C(=C/C=C/C(C)=C(/F)C(=O)O)c1cc(C(C)(C)C)cc(C(C)(C)C)c1OCC(F)F | active |
| C/C(=C/C=C(F)/C(C)=C/C(=O)O)c1cc(C(C)(C)C)cc(C(C)(C)C)c1OCC(F)F | active |
| CC(/C=C/C(F)=C(/C)c1cc(C(C)(C)C)cc(C(C)(C)C)c1OCC(F)F)=C\C(=O)O | active |
| CC(/C=C/C1(c2cc3c(cc2C)C(C)(C)CCC3(C)C)CC1)=C\C(=O)O | active |
| CC(/C=C/C1(c2ccc3c(c2)C(C)(C)CCC3(C)C)CC1)=C\C(=O)O | active |
| CC(/C=C/C1(c2cc3c(cc2C)C(C)(C)CCC3(C)C)CCCC1)=C\C(=O)O | active |
| CC(/C=C/C1(c2ccc3c(c2)C(C)(C)CCC3(C)C)CCCC1)=C\C(=O)O | active |
| CC(/C=C/C1=C(c2cc3c(cc2C)C(C)(C)CCC3(C)C)CCC1)=C\C(=O)O | active |
| CC(/C=C\C1=C(c2ccc3c(c2)C(C)(C)CCC3(C)C)CCC1)=C\C(=O)O | active |
| CC(/C=C\C1CC1(C)c1cc2c(cc1C)C(C)(C)CCC2(C)C)=C\C(=O)O | active |
| CC(/C=C/C1CC1(C)c1ccc2c(c1)C(C)(C)CCC2(C)C)=C\C(=O)O | active |
| C/C(=C/C=C/C(C)=C/C(=O)O)c1ccc2c(c1)C(C)(C)CO2 | active |
| C/C(=C/C=C/C(C)=C/C(=O)O)c1ccc2c(c1)C(C)(C)CCO2 | active |
| C/C(=C/C=C/C(C)=C/C(=O)O)c1ccc2c(c1)C(C)(C)CS2 | active |
| C/C(=C/C=C/C(C)=C/C(=O)O)c1ccc2c(c1)C(C)(C)CCS2 | active |
| C/C(=C/C=C/C(C)=C/C(=O)O)c1cc(C(C)(C)C)cc(C(C)(C)C)c1OCC(F)F | active |
| C/C(=C/C=C\C(C)=C\C(=O)O)c1cc(C(C)(C)C)cc(C(C)(C)C)c1OCCCF | active |
| CC(/C=C/CC1(c2cc(C(C)(C)C)cc(C(C)(C)C)c2)CC1)=C\C(=O)O | active |
| CC(/C=C/CC1(c2cc(C(C)(C)C)cc3c2CCC3(C)C)CC1)=C\C(=O)O | active |
| CC(/C=C/CC1(c2cc3c(cc2C)C(C)(C)CCC3(C)C)CC1)=C\C(=O)O | active |
| CC(/C=C/CC1(c2ccc3c(c2)C(C)(C)CCC3(C)C)CC1)=C\C(=O)O | active |
| CC(/C=C/CC1(c2ccc3c(c2)C(C)(C)CCC3(C)C)CCCC1)=C\C(=O)O | active |
| CC(/C=C/CN(CC1CC1)c1cc(C(C)(C)C)cc2c1CCC2(C)C)=C\C(=O)O | active |
| C/C(=C/C(=O)O)c1cc2c(-c3cc(C(C)(C)C)cc(C(C)(C)C)c3C)cccc2s1 | active |
| C/C(=C/C(=O)O)c1ccc2[nH]cc(-c3cc(C(C)(C)C)cc(C(C)(C)C)c3OCC(F)F)c2c1 | active |
| C/C(=C/C(=O)O)c1cc2c(o1)CCCC2c1ccc2c(c1)C(C)(C)CCC2(C)C | active |
| CC1=C(/C=C/C(C)=C/C=C/C(C)=C\C(=O)O)C(C)(C)CCC1 | active |
| C/C(=C/c1cc(C(=O)O)cs1)c1cc2c(cc1C)C(C)(C)CCC2(C)C | active |
| CC1=C(CC/C(C)=C\c2ccc(C(=O)O)cc2)C(C)(C)CCC1 | active |
| C/C(=C/c1ccc(C(=O)O)cc1)c1cc2c(cc1Br)C(C)(C)CCC2(C)C | active |
| C/C(=C/c1ccc(C(=O)O)cc1)c1cc2c(cc1Cl)C(C)(C)CCC2(C)C | active |
| C/C(=C/c1ccc(C(=O)O)cn1)c1cc2c(cc1C)C(C)(C)CCC2(C)C | active |
| C/C(=C/c1ccc(C(=O)O)s1)c1cc2c(cc1C)C(C)(C)CCC2(C)C | active |
| C/C(=C/c1ccc(C(=O)O)s1)c1ccc2c(c1)C(C)(C)CCC2(C)C | active |
| C/C(=C/c1csc(C(=O)O)c1)c1cc2c(cc1C)C(C)(C)CCC2(C)C | active |
| C/C(=C/c1nc(C(=O)O)c[nH]1)c1cc2c(cc1C)C(C)(C)CCC2(C)C | active |
| C/C(=C/c1ncc(C(=O)O)s1)c1cc2c(cc1C)C(C)(C)CCC2(C)C | active |
| C/C(=C/c1ncc(C(=O)O)s1)c1ccc2c(c1)C(C)(C)CCC2(C)C | active |
| C/C(=C/c1ccc(C(=O)O)o1)c1cc2c(cc1C)C(C)(C)CCC2(C)C | active |
| CC(=C/C(=O)O)/C=C/C=C(C)/C=C1\CCCCc2ccccc21 | active |
| CC(=C/C(=O)O)/C=C/C=C(C)/C=C1\CCCc2c(C)cccc21 | active |
| CC(=C/C(=O)O)/C=C/C=C(C)/C=C1\CCCc2cc(C)ccc21 | active |
| CC(=C/C(=O)O)/C=C/C=C(C)/C=C1\CCCc2ccc(C)cc21 | active |
| CC(=C/C(=O)O)/C=C/C=C(C)/C=C1\CCCc2cccc(C)c21 | active |
| CC(=C/C(=O)O)/C=C/C=C(C)/C=C1\CCCc2ccccc21 | active |
| CC1=C(/C=C/C(C)=C/C=C/C(C)=C\C(=O)O)C(C)(C)CCC1 | active |
| C/C(=C/c1ccc(C(=O)O)cc1)c1ccc2c(c1)C(C)(C)CCC2(C)C | active |
| C/C(=C/c1ccc2c(c1)C(C)(C)CCS2)c1ccc(C(=O)O)cc1 | active |
| C/C(=C/c1ccc2c(c1)C(C)(C)CCC2(C)C)c1ccc(C(=O)O)cc1 | active |
| C/C=C(/c1ccc(C(=O)O)cc1)c1ccc2c(c1)C(C)(C)CCC2(C)C | active |
| C/C=C(/c1ccc(C(=O)O)cc1)c1ccc2c(c1)C(C)(C)CCC2(C)C | active |
| C=C(c1ccc(C(=O)O)cc1)c1cc2c(cc1C)CCCC2(C)C | active |
| C=C(c1ccc(C(=O)O)cc1)c1cc2c(cc1C)C(C)(C)CCC2 | active |
| C=C(c1ccc(C(=O)NO)cc1)c1cc2c(cc1C)C(C)(C)CCC2(C)C | active |
| C=C(c1ccc(C(=O)O)cn1)c1cc2c(cc1C)C(C)(C)CCC2(C)C | active |
| C=C(c1ccc(C(=O)O)s1)c1cc2c(cc1C)C(C)(C)CCC2(C)C | active |
| Cc1cc2c(cc1C(C)(C)c1ccc(C(=O)O)cc1)C(C)(C)CCC2(C)C | active |
| Cc1cc2c(cc1C1(c3ccc(C(=O)O)cc3)CC1)C(C)(C)CCC2(C)C | active |
| Cc1cc2c(cc1C1(c3ccc(C(=O)O)cn3)CC1)C(C)(C)CCC2(C)C | active |
| Cc1cc2c(cc1C1(c3ccc(C(=O)O)cc3)CO1)C(C)(C)CCC2(C)C | active |
| Cc1cc2c(cc1Cc1ccc(C(=O)O)cc1)C(C)(C)CCC2(C)C | active |
| Cc1cc2c(cc1Oc1ccc(C(=O)O)cc1)C(C)(C)CCC2(C)C | active |
| Cc1cc2c(cc1S(=O)(=O)c1ccc(C(=O)O)cc1)C(C)(C)CCC2(C)C | active |
| Cc1cc2c(cc1Sc1ccc(C(=O)O)cc1)C(C)(C)CCC2(C)C | active |
| Cc1cc2c(cc1Sc1ccc(C(=O)O)cn1)C(C)(C)CCC2(C)C | active |
| Cc1cc2c(cc1Sc1ncc(C(=O)O)s1)C(C)(C)CCC2(C)C | active |
| Cc1cc2c(cc1/C(=N/O)c1ccc(C(=O)O)cc1)C(C)(C)CCC2(C)C | active |
| Cc1cc2c(cc1/C(=N/O)c1ccc(C(=O)O)cc1)C(C)(C)CCC2(C)C | active |
| Cc1cc2c(cc1/C(=N/OCC#N)c1ccc(C(=O)O)cc1)C(C)(C)CCC2(C)C | active |
| C=CCO/N=C(\c1ccc(C(=O)O)cc1)c1cc2c(cc1C)C(C)(C)CCC2(C)C | active |
| Cc1cc2c(cc1/C(=N/OCCN)c1ccc(C(=O)O)cc1)C(C)(C)CCC2(C)C | active |
| Cc1cc2c(cc1-n1c(C)nc3cc(C(=O)O)ccc31)C(C)(C)CCC2(C)C | active |
| Cc1cc2c(cc1-n1c(N)nc3cc(C(=O)O)ccc31)C(C)(C)CCC2(C)C | active |
| Cc1cc2c(cc1-n1c(C(F)(F)F)nc3cc(C(=O)O)ccc31)C(C)(C)CCC2(C)C | active |
| Cc1cc2c(cc1-n1ccc3cc(C(=O)O)ccc31)C(C)(C)CCC2(C)C | active |
| Cc1cc2c(cc1-n1cnc3cc(C(=O)O)ccc31)C(C)(C)CCC2(C)C | active |
| Cc1cc2c(cc1-n1nnc3cc(C(=O)O)ccc31)C(C)(C)CCC2(C)C | active |
| Cc1oc(/C=C/C(=O)O)cc1Cc1ccc2c(c1)C(C)(C)CCC2(C)C | active |
| COC(=O)c1ccc2c3c(n(CCCN(C)C)c(=O)c2c1)-c1ccccc1C3=O | active |
| NCCCn1c2c(c3ccc(N)cc3c1=O)Cc1ccccc1-2 | active |
| NCCCCCCn1c2c(c3ccccc3c1=O)C(=O)c1ccccc1-2 | active |
| NCCCCCn1c2c(c3ccccc3c1=O)C(=O)c1ccccc1-2 | active |
| NCCCCn1c2c(c3ccccc3c1=O)C(=O)c1ccccc1-2 | active |
| NCCCn1c2c(c3ccc(N)cc3c1=O)C(=O)c1ccccc1-2 | active |
| NCCCn1c2c(c3ccc(NCC(=O)O)cc3c1=O)C(=O)c1ccccc1-2 | active |
| NCCCn1c2c(c3ccccc3c1=O)C(=O)c1ccccc1-2 | active |
| NCCn1c2c(c3ccccc3c1=O)C(=O)c1ccccc1-2 | active |
| CC(C)=CCN(C/C=C/C(C)=C/C(=O)O)c1cc(C(C)(C)C)cc2c1CCC2(C)C | inactive |
| CC(=O)Nc1ccc(/C=C2\C(C)=C(CC(=O)O)c3cc(F)ccc32)cc1 | inactive |
| CC(C)(C)c1cc(C2=Cc3ccc(C(=O)O)cc3OC2)cc(C(C)(C)C)c1O | inactive |
| CC(C)(C)c1cc(C2=Cc3ccc(C(=O)O)cc3OC2)ccc1O | inactive |
| CC(C)(C)c1cc(-c2ccc3cc(C(=O)O)ccc3c2)ccc1O | inactive |
| CC1=C(CC(N)=O)c2cc(F)ccc2/C1=C/c1ccc(C(C)C)cc1 | inactive |
| CC1=C(CC(=O)O)c2cc(C)ccc2/C1=C/c1ccc(C(C)C)cc1 | inactive |
| CC1=C(CC(=O)O)c2cc(Cl)ccc2/C1=C/c1ccc(C(C)C)cc1 | inactive |
| CC1=C(CC(=O)O)c2cc(C(C)C)ccc2/C1=C/c1ccc(C(C)C)cc1 | inactive |
| CC1=C(CC(=O)O)c2ccccc2/C1=C/c1ccc(C(C)C)cc1 | inactive |
| CC1=C(CCC#N)c2cc(F)ccc2/C1=C/c1ccc(C(C)C)cc1 | inactive |
| CC1=C(CC(N)=O)c2cc(F)ccc2/C1=C/c1ccc(C(C)C)cc1 | inactive |
| CC1(C)CC=C(C#Cc2ccccc2)c2ccc(C(=O)/C=C/c3ccc(C(=O)O)cc3)cc21 | inactive |
| CC1(C)CC=C(c2ccccc2)c2ccc(C(O)C(=O)Nc3ccc(C(=O)O)cc3F)cc21 | inactive |
| CC1(C)CCC(C)(C)c2cc(NC(=O)c3ccc(C(=O)O)cc3)ccc21 | inactive |
| CC1(C)CCC(C)(C)c2cc(/C=C/c3ccc(C(=O)O)cc3)ccc21 | inactive |
| CC1(C)CCC(C)(C)c2cc(C3=Cc4ccc(C(=O)O)cc4OC3)ccc21 | inactive |
| CC1(C)CCC(C)(C)c2cc(C3COc4cc(C(=O)O)ccc4C3)ccc21 | inactive |
| CC1(C)CCC(C)(C)c2cc(-c3ccc4cc(C(=O)O)ccc4c3)ccc21 | inactive |
| CC1(C)CCC(C)(C)c2cc(-c3cc(C(=O)O)no3)ccc21 | inactive |
| CC1(C)CCC(C)(C)c2cc3c(cc21)OC1CCCCC31c1ccc(O)cc1 | inactive |
| CC12CCCc3cc(NC(=O)c4ccc(C(=O)O)cc4)cc(c31)CCC2 | inactive |
| CC12CCCc3cc(NC(=O)c4ccc(C(=O)O)cn4)cc(c31)CCC2 | inactive |
| CC12CCCc3cc(NC(=O)c4ccc(C(=O)O)nc4)cc(c31)CCC2 | inactive |
| CC12CCCc3cc(NC(=O)c4ccc(C(=O)O)s4)cc(c31)CCC2 | inactive |
| CC12CCCc3cc(C(=O)Nc4ccc(C(=O)O)c(Cl)c4)cc(c31)CCC2 | inactive |
| CC12CCCc3cc(C(=O)Nc4ccc(C(=O)O)c(F)c4)cc(c31)CCC2 | inactive |
| CC12CCCc3cc(C(=O)Nc4ccc(C(=O)O)c(O)c4)cc(c31)CCC2 | inactive |
| CC12CCCc3cc(C(=O)Nc4ccc(C(=O)O)cc4)cc(c31)CCC2 | inactive |
| CC12CCCc3cc(C(=O)Nc4ccc(C(=O)O)cn4)cc(c31)CCC2 | inactive |
| CC12CCCc3cc(C(=O)Nc4ccc(C(=O)O)nc4)cc(c31)CCC2 | inactive |
| CC12CCCc3cc(C(=O)Nc4ccc(C(=O)O)s4)cc(c31)CCC2 | inactive |
| CC1=C(CC(=O)O)c2cc(F)ccc2/C1=C\c1ccc(C#N)cc1 | inactive |
| CC1=C(CC(=O)O)c2cc(F)ccc2/C1=C\c1ccc(C(C)(C)C)cc1 | inactive |
| CC1=C(CC(=O)O)c2cc(F)ccc2/C1=C\c1ccc(-c2ccccn2)cc1 | inactive |
| CC1=C(CC(=O)O)c2cc(F)ccc2/C1=C\c1cccc(C#N)c1 | inactive |
| Cc1cc(=O)oc2c(/C=N/NC(=O)CSc3nc4ccccc4s3)c(O)ccc12 | inactive |
| Cc1cc2c(cc1-c1cc(C(=O)O)no1)C(C)(C)C(C)CC2(C)C | inactive |
| CC1c2ccccc2N=C(c2ccc(C(=O)O)cc2)c2cc3c(cc21)C(C)(C)CCC3(C)C | inactive |
| CCC(CC)(c1ccc(OCC(=O)O)c(C)c1)c1ccc(OCc2c(-c3c(Cl)cccc3Cl)noc2C(C)C)c(C)c1 | inactive |
| CCC(Oc1ccc(C2(c3ccc(OC(CC)C(=O)O)c(C)c3)CCCCC2)cc1C)C(=O)O | inactive |
| CCCCCCCN(c1ccc(C(=O)O)cc1)c1ccc2c(c1)C(C)(C)CCC2(C)C | inactive |
| CCCCCCc1cc(OCOCCOC)c(C23CC4CC(CC(C4)C2)C3)cc1C(=O)/C=C/c1ccc(C(=O)O)cc1 | inactive |
| CCCCCNC(=O)N1CCC(CN(Cc2ccc(Cl)cc2)Cc2ccc([N+](=O)[O-])s2)C1 | inactive |
| CCCCCOc1cc2c(cc1-c1cc(/C=C/C(=O)O)ccc1O)C(C)(C)CCC2(C)C | inactive |
| CCCCCc1cc(OCOCCOC)c(C23CC4CC(CC(C4)C2)C3)cc1C(=O)/C=C/c1ccc(C(=O)O)cc1 | inactive |
| CCCCc1cc(OCOCCOC)c(C23CC4CC(CC(C4)C2)C3)cc1C(=O)/C=C/c1ccc(C(=O)O)cc1 | inactive |
| CCCOc1cc2c(cc1C(=C1CC1)c1ccc(C(=O)O)cc1)C(C)(C)CCC2(C)C | inactive |
| CCCc1cc(OCOCCOC)c(C23CC4CC(CC(C4)C2)C3)cc1C(=O)/C=C/c1ccc(C(=O)O)cc1 | inactive |
| CCN(c1ccc(C(=O)O)cn1)c1cc(OCC(C)C)c(C(C)C)cc1Nc1ccccc1 | inactive |
| CCN(c1ccc(C(=O)O)cn1)c1cc(OCC(C)C)c(C(C)C)cc1/C=C/c1ccccc1 | inactive |
| CCN(c1ccc(C(=O)O)cn1)c1cc(OCC(C)C)c(C(C)C)cc1-c1ccccc1 | inactive |
| CCN(c1ccc(C(=O)O)cn1)c1ccc2c(C)cc(=O)n(C)c2n1 | inactive |
| CCN(c1ccc2c(c1)C(C)(C)CCC2(C)C)c1ccc(P(=O)(O)O)cn1 | inactive |
| CCOC(=O)CNc1ccc2c3c(n(CCCN)c(=O)c2c1)-c1ccccc1C3=O | inactive |
| CCOC(=O)N1CCC(CN(Cc2ccc(Cl)cc2)Cc2ccc([N+](=O)[O-])s2)C1 | inactive |
| CCOc1c(F)c(F)c(CN(c2ccc(C(=O)O)cc2)c2ccc3c(c2)C(C)(C)CCC3(C)C)c(F)c1F | inactive |
| CCOc1ccc(/C=C2\C(C)=C(CC(=O)O)c3cc(F)ccc32)cc1 | inactive |
| CCS(=O)(=O)c1ccc(CC(=O)Nc2nc(-c3cccc(Cl)c3)c(C(=O)c3ccccc3Cl)s2)cc1 | inactive |
| CCc1cc(OCOCCOC)c(C23CC4CC(CC(C4)C2)C3)cc1C(=O)/C=C/c1ccc(C(=O)O)cc1 | inactive |
| C=C1c2ccccc2C(=O)N(C)c2ccc(CC)cc21 | inactive |
| CCc1ccc2c(c1)C(CC(=O)O)=C(C)/C2=C\c1ccc(C(C)C)cc1 | inactive |
| CC1=C(CC(=O)O)c2cc(F)ccc2/C1=C/c1ccc(N(C)C)cc1 | inactive |
| CN(CCOc1ccc(CC2SC(=O)NC2=O)cc1)c1ccccn1 | inactive |
| COC(=O)c1ccc(/C=C(/C)c2ccc3c(c2)C(C)(C)CO3)cc1 | inactive |
| COC(=O)c1ccc(/C=C(/C)c2ccc3c(c2)C(C)(C)CCO3)cc1 | inactive |
| COC(=O)c1ccc(/C=C(/C)c2ccc3c(c2)C(C)(C)CCS3)cc1 | inactive |
| C#CCc1cc(OCOCCOC)c(C23CC4CC(CC(C4)C2)C3)cc1C(=O)/C=C/c1ccc(C(=O)O)cc1 | inactive |
| C=CCc1cc(OCOCCOC)c(C23CC4CC(CC(C4)C2)C3)cc1C(=O)/C=C/c1ccc(C(=O)O)cc1 | inactive |
| COCCOCOc1cc(C/C=C/c2ccccc2)c(C(=O)/C=C/c2ccc(C(=O)O)cc2)cc1C12CC3CC(CC(C3)C1)C2 | inactive |
| COCCOCOc1cc(Cc2ccccc2)c(C(=O)/C=C/c2ccc(C(=O)O)cc2)cc1C12CC3CC(CC(C3)C1)C2 | inactive |
| COCCOCOc1cc(O)c(C(=O)/C=C/c2ccc(C(=O)O)cc2)cc1C12CC3CC(CC(C3)C1)C2 | inactive |
| COCCOCOc1ccc(C(=O)/C=C/c2ccc(C(=O)O)cc2)cc1C12CC3CC(CC(C3)C1)C2 | inactive |
| COc1cc(NC(=O)c2cc3c4c(c2)CCCC4(C)CCC3)ccc1C(=O)O | inactive |
| COc1cc(NC(=O)c2cc3c4c(c2)CCCC4CCC3)ccc1C(=O)O | inactive |
| COc1cc(NC(=O)c2cc3c4c(c2)CCCCC4CCCC3)ccc1C(=O)O | inactive |
| COc1cc(/C=C2\C(=O)N(c3cccc([N+](=O)[O-])c3)N=C2c2ccccc2)ccc1O | inactive |
| COc1ccc(/C=C2\C(C)=C(CC(=O)O)c3cc(F)ccc32)cc1 | inactive |
| COc1ccc(-c2ccc3cc(C(=O)O)ccc3c2)cc1C12CC3CC(CC(C3)C1)C2 | inactive |
| COc1ccc2c(C)cc(=O)oc2c1/C=N/NC(=O)CSc1nc2ccccc2s1 | inactive |
| COc1ccc2c(c1)C(CC(=O)O)=C(C)/C2=C\c1ccc(C(C)C)cc1 | inactive |
| COc1cccc(/C=C2\C(C)=C(CC(=O)O)c3cc(F)ccc32)c1 | inactive |
| CC1=C(CC(=O)O)c2cc(F)ccc2/C1=C/c1ccc([S@](C)=O)cc1 | inactive |
| CC(/C=C/C(=O)N(Cc1ccccc1)c1cc(C(C)(C)C)cc(C(C)(C)C)c1)=C\C(=O)O | inactive |
| CC(/C=C/C(=O)Nc1cc(C(C)(C)C)cc(C(C)(C)C)c1)=C\C(=O)O | inactive |
| CC(/C=C/CN(Cc1ccccc1)c1cc(C(C)(C)C)cc(C(C)(C)C)c1)=C\C(=O)O | inactive |
| CC(/C=C/CNc1cc(C(C)(C)C)cc(C(C)(C)C)c1)=C\C(=O)O | inactive |
| C/C(=C/c1ccc(C(=O)O)cc1)c1ccc2c(c1)C(C)(C)CO2 | inactive |
| C/C(=C/c1ccc(C(=O)O)cc1)c1ccc2c(c1)C(C)(C)CCO2 | inactive |
| C/C(=C/c1ccc(C(=O)O)cc1)c1ccc2c(c1)C(C)(C)CS2 | inactive |
| C/C(=C/c1ccc2c(c1)C(C)(C)CCC2(C)C)c1ccc(C(=O)O)cc1 | inactive |
| C/C(=C/c1cc(C(=O)O)no1)c1ccc2c(c1)C(C)(C)CCC2(C)C | inactive |
| C/C(=C/c1cc(C(=O)O)no1)c1ccc2c(c1)C(C)(C)CCC2(C)C | inactive |
| Cc1cc2c(cc1/C=C/c1ccc(C(=O)O)cc1)C(C)(C)CCC2(C)C | inactive |
| Cc1cc2c(cc1-c1cc(C(=O)O)no1)C(C)(C)CCC2(C)C | inactive |
| Cc1ccc(C2=CCC(C)(C)c3cc(C(O)C(=O)Nc4ccc(C(=O)O)cc4F)ccc32)cc1 | inactive |
| O=C(O)c1ccc(NC(=O)c2cc3c4c(c2)CCCC4CCC3)cc1 | inactive |
| O=C(Nc1ccc(C(=O)O)c(Cl)c1)c1cc2c3c(c1)CCCC3CCC2 | inactive |
| O=C(Nc1ccc(C(=O)O)c(F)c1)c1cc2c3c(c1)CCCC3CCC2 | inactive |
| O=C(Nc1ccc(C(=O)O)c(O)c1)c1cc2c3c(c1)CCCC3CCC2 | inactive |
| O=C(Nc1ccc(C(=O)O)nc1)c1cc2c3c(c1)CCCC3CCC2 | inactive |
| O=C(O)c1ccc(NC(=O)c2cc3c4c(c2)CCCC4CCC3)nc1 | inactive |
| O=C(Nc1ccc(C(=O)O)s1)c1cc2c3c(c1)CCCC3CCC2 | inactive |
| O=C(O)c1ccc(NC(=O)c2cc3c4c(c2)CCCCC4CCCC3)cc1 | inactive |
| O=C(Nc1ccc(C(=O)O)c(Cl)c1)c1cc2c3c(c1)CCCCC3CCCC2 | inactive |
| O=C(Nc1ccc(C(=O)O)c(F)c1)c1cc2c3c(c1)CCCCC3CCCC2 | inactive |
| O=C(Nc1ccc(C(=O)O)c(O)c1)c1cc2c3c(c1)CCCCC3CCCC2 | inactive |
| O=C(O)c1ccc(NC(=O)c2cc3c4c(c2)CCCCC4CCCC3)nc1 | inactive |
| O=C(O)c1ccc(NC(=O)c2cc3cc(c2)CCCCCCCCCC3)cc1 | inactive |
| O=C(O)c1ccc(C(=O)Nc2cc3c4c(c2)CCCC4CCC3)cc1 | inactive |
| O=C(O)c1ccc(C(=O)Nc2cc3c4c(c2)CCCCC4CCCC3)cc1 | inactive |
| O=C(O)c1ccc(C(=O)Nc2cc3cc(c2)CCCCCCCCCC3)cc1 | inactive |
| O=C(Nc1cc2c3c(c1)CCCC3CCC2)c1ccc(C(=O)O)nc1 | inactive |
| O=C(O)c1ccc(C(=O)Nc2cc3c4c(c2)CCCC4CCC3)nc1 | inactive |
| O=C(O)c1ccc(C(=O)Nc2cc3c4c(c2)CCCCC4CCCC3)nc1 | inactive |
| O=C(O)c1ccc(C(=O)Nc2cc3c4c(c2)CCCC4CCC3)s1 | inactive |
| C/C(=C/C=C/C(C)=C/C(=O)O)c1cc(-c2cccc(F)c2)cc(C(C)C)c1OCC(F)F | queries |
| C/C(=C/C=C/C(C)=C/C(=O)O)c1cc(-c2ccc(F)cc2)cc(C(C)C)c1OCC(F)F | queries |
| CCN1C(=O)CC(C)(C)c2cc(C)c(-c3cc(/C=C4\SC(=O)NC4=O)ccc3OC(F)(F)F)cc21 | queries |
| C/C(=C/C=C/C(C)=C/C(=O)O)c1cc(C(C)(C)C)cc(C(C)(C)C)c1OCC(F)F | queries |
| C/C(=C/C=C/C(C)=C/C(=O)O)c1cc(C(C)C)cc(C(C)C)c1OCC(F)F | queries |
| CC1=C(/C=C/C(C)=C\C=C\C(C)=C\C(=O)O)C(C)(C)CCC1 | queries |
| COc1c(/C(C)=C\C=C\C(C)=C\C(=O)O)cc(C(C)(C)C)cc1C(C)(C)C | queries |
| COc1c(/C(C)=C\C=C\C(C)=C\C(=O)O)cc(C(C)C)cc1C(C)C | queries |
| CC(/C=C/C1CC1(C)c1ccc2c(c1)C(C)(C)CCC2(C)C)=C\C(=O)O | queries |
| C/C(=N/[N-]C(=S)Nc1ccc(C(C)C)cc1)c1ccc(-c2ccccc2)cc1 | hits |
| Cc1ccc(NC2=NC(=O)/C(=C/c3ccc(O)cc3)S2)cc1C | hits |
| CCOc1ccc(NC(=S)[N-]/N=C(/C)c2cccc(F)c2)cc1 | hits |
| O=C([O-])CCc1ccc(S(=O)(=O)N2CCc3ccc(Cl)cc32)cc1 | hits |
| CCCC/C(C)=N/NC(=O)c1ccc(CSc2ccc(Cl)cc2)cc1 | hits |
| CC(C)(C)c1ccc(/C=C(/C#N)C(=O)Nc2cccc(C(F)(F)F)c2)cc1 | hits |
| O=C(NC(CCn1cncn1)c1ccccc1)C1(c2ccc(F)cc2)CC1 | hits |
| CC(NC(=O)c1cccc(OCc2ccccc2)c1)c1ccccc1 | hits |
| OC(c1ccccc1)c1nc2ccccc2n1CCCCOc1ccccc1 | hits |
| CC(C)(C)c1ccc(/C=N/[N-]C(=S)NCc2ccccc2)cc1 | hits |
| CC(C)Cc1cc(C(=O)NC(c2ccc3c(c2)CCO3)C2CC(O)C2)nc(=O)[nH]1 | hits |
| CC(C)(C)c1cc(/C=N/NC(=O)c2ccc(N)cc2)cc(C(C)(C)C)c1 | hits |
| Cc1ccc(C(=O)Nc2ccc(C(=O)/C=C/c3cccs3)cc2)cc1 | hits |
| Cc1ccc(-n2nnnc2SCc2ccc(C#N)cc2)c(C)c1 | hits |
| O=C([O-])CCc1ccc(S(=O)(=O)N2CCc3ccc(F)cc32)cc1 | hits |
| COc1ccc(CCNC(=S)NC23CC4CC(CC(C4)C2)C3)cc1 | hits |
| Cn1c2c(cc(C(=O)N3CCC(Cc4ccc(CO)cc4)C3)c1=O)CCCC2 | hits |
| CC/C(=N/[N-]C(=S)Nc1ccc(Cl)cc1Cl)c1ccc(O)cc1 | hits |
| Cc1cc(C)c(NC(=O)CCCSCCOc2ccc(C(C)C)c(C)c2)c(C)c1 | hits |
| CC(C)Oc1ccc(NC(=O)c2ccccc2CCc2ccccc2)cc1 | hits |

**Supplementary Table 3.** Primers utilized for target gene quantification (quantitative real-time PCR).

| gene | forward primer (5’ -> 3’) | reverse primer (5’ -> 3’) |
| --- | --- | --- |
| GAPDH | ATA TGA TTC CAC CCA TGG CA | GAT GAT GAC CCT TTT GGC TC |
| ABCA1 | TTC GCT CTG AGA TGA GCA CCA | TTT CAA GCG GGC ATA GAA CCA |
| ANGPTL4 | CGT ACC CTT CTC CAC TTG GG | GCT CTT GGC CGA GTT CTT G |
| ApoE | GGT CGC TTT TGG GAT TAC CT | CTC CAG TTC CGA TTT GT |

**References**

1. Schmidt, J. *et al.* A dual modulator of farnesoid X receptor and soluble epoxide hydrolase to counter nonalcoholic steatohepatitis. *J. Med. Chem.* **60,** 7703–7724 (2017).

2. Heitel, P., Achenbach, J., Moser, D., Proschak, E. & Merk, D. DrugBank screening revealed alitretinoin and bexarotene as liver X receptor modulators. *Bioorg Med Chem Lett* **27,** 1193–1198 (2017).

3. Flesch, D. *et al.* Non-acidic farnesoid X receptor modulators. *J. Med. Chem.* **60,** 7199–7205 (2017).
